# DMRT1-mediated regulation of *TOX3* modulates expansion of the gonadal steroidogenic cell lineage in the chicken embryo

**DOI:** 10.1242/dev.201466

**Published:** 2023-03-09

**Authors:** Martin A. Estermann, Andrew T. Major, Craig A. Smith

**Affiliations:** Department of Anatomy and Developmental Biology, Monash Biomedicine Discovery Institute, Monash University, Clayton, VIC 3800, Australia

**Keywords:** DMRT1, Gonad, Steroidogenic, TOX3, Chicken

## Abstract

During gonadal sex determination, the supporting cell lineage differentiates into Sertoli cells in males and pre-granulosa cells in females. Recently, single cell RNA-seq data have indicated that chicken steroidogenic cells are derived from differentiated supporting cells. This differentiation process is achieved by a sequential upregulation of steroidogenic genes and downregulation of supporting cell markers. The exact mechanism regulating this differentiation process remains unknown. We have identified *TOX3* as a previously unreported transcription factor expressed in embryonic Sertoli cells of the chicken testis. *TOX3* knockdown in males resulted in increased *CYP17A1*-positive Leydig cells. *TOX3* overexpression in male and female gonads resulted in a significant decline in *CYP17A1*-positive steroidogenic cells. *In ovo* knockdown of the testis determinant *DMRT1* in male gonads resulted in a downregulation of TOX3 expression. Conversely, DMRT1 overexpression caused an increase in *TOX3* expression. Taken together, these data indicate that DMRT1-mediated regulation of *TOX3* modulates expansion of the steroidogenic lineage, either directly, via cell lineage allocation, or indirectly, via signaling from the supporting to steroidogenic cell populations.

## Teaser

**Summary:** TOX3 overexpression and knockdown studies reveal that it modulates differentiation of the steroidogenic cell population and that its dysregulation may underlie increased steroidogenic capacity, which might lead to polycystic ovarian syndrome.

## INTRODUCTION

During early embryonic development, the gonads typically differentiate into either testes or ovaries. In mammals, the testicular developmental pathway is initiated by the expression of the Y-linked gene *Sry* in the supporting cells, triggering the upregulation of thousands of genes over a short period of time and resulting in the differentiation of the supporting cells into Sertoli cells (Koopman, 1999; Stevant et al., 2018; Koopman et al., 1991). One of the transcriptional targets of Sry is *Sox9*, a SRY-related HMG box family member, which is crucial in Sertoli differentiation (Koopman, 1999; Sekido, 2010; Vidal et al., 2001; Hiramatsu et al., 2009; Bullejos and Koopman, 2005). Sertoli cells then direct differentiation of other testicular cell types (Habert et al., 2001). In the absence of this masculinizing signal, gonads differentiate into ovaries through the stabilization of the Rspo1/Wnt/β-catenin signaling pathway (Chassot et al., 2014; Pannetier et al., 2016; Chassot et al., 2008).

In the chicken model, the molecular trigger for Sertoli cell differentiation is the Z-chromosome linked gene *DMRT1* (Smith et al., 2009a). *DMRT1* is unrelated to *Sry*, and it encodes a zinc-finger-like transcription factor (Raymond et al., 2000; Shetty et al., 2002; Koopman and Loffler, 2003; Koopman, 2009). Despite the differing gonadal sex determination triggers in mammals and birds, the genetic regulation of gonadal development and sexual differentiation is largely conserved. This includes the supporting cell markers *DMRT1*, *AMH* and *SOX9* in the avian testis, and the markers *WNT4*, *RSPO1*, aromatase (*CYP19A1*) and *FOXL2* in the ovary (Major et al., 2019; Lambeth et al., 2013, 2014, 2016; Smith et al., 2008b). Recently, chicken single-cell RNA-seq data indicates that, although gonadal cell types are conserved, their developmental origin is not (Sekido and Lovell-Badge, 2007; Estermann et al., 2020). In the mouse embryo, the supporting cell lineage derives from the coelomic epithelium (Stevant and Nef, 2019; Lin et al., 2017; Karl and Capel, 1998). In chicken, the coelomic epithelium gives rise to gonadal epithelium and interstitial cells (Estermann et al., 2020). The supporting cells derive from a *DMRT1*-, *PAX2*-, *OSR1*- and *WNT4*-positive pre-existing mesenchymal population (Estermann et al., 2020). Additionally, the scRNA-seq data strongly suggest that the steroidogenic cells derive from differentiating supporting cells (Sertoli and pre-granulosa cells) (Estermann et al., 2020). This differentiation process is achieved by a sequential upregulation of steroidogenic genes, resulting in cells expressing both steroidogenic and Sertoli cell markers (intermediate cells), and followed by the downregulation of supporting cell markers (Estermann et al., 2020). Owing to the novelty of this discovery in the chicken, the exact mechanism regulating this differentiation process remains unknown.

To expand our understanding of normal and aberrant gonadal development and differentiation, it is essential to identify novel regulators of ovarian and testicular development. Our laboratory has already identified *TOX3* (TOX high mobility group box family member 3) as a gene previously unreported to be expressed in chicken Sertoli cells (Estermann et al., 2020). In mouse, *Tox3* is a high mobility group (HMG) box transcription factor predominantly expressed in the brain, where it plays a protective role inducing an anti-apoptotic response, interacting with CBP and/or CREB or CBP and/or CITED1 (Dittmer et al., 2011; Sahu et al., 2016). TOX3 acts as a transcriptional activator upregulating (directly or indirectly) a large number of genes involved cell proliferation, migration, mammary gland development and breast cancer (Seksenyan et al., 2015; Jiang et al., 2019). Additionally, DNA variants in the *TOX3* locus have been associated with polycystic ovarian syndrome (PCOS) in several human populations, a syndrome associated with higher levels of androgens, (Tian et al., 2020; Bakhashab and Ahmed, 2019; Pau et al., 2017; Liu et al., 2020). However, the exact functional mechanism of *TOX3* in this disease or in the gonadal context is unclear. In this study we characterized the expression pattern of *TOX3* in the developing chicken gonad, focusing on how it is regulated and its potential role in gonadal supporting and steroidogenic cell differentiation. Our data support a model in which TOX3 modulates differentiation of the steroidogenic cell population, and its dysregulation may underlie increased steroidogenic capacity, leading to PCOS.

## RESULTS

### TOX3 is expressed in a subset of Sertoli cells after the onset of gonadal sex differentiation

**Fig. 1.**
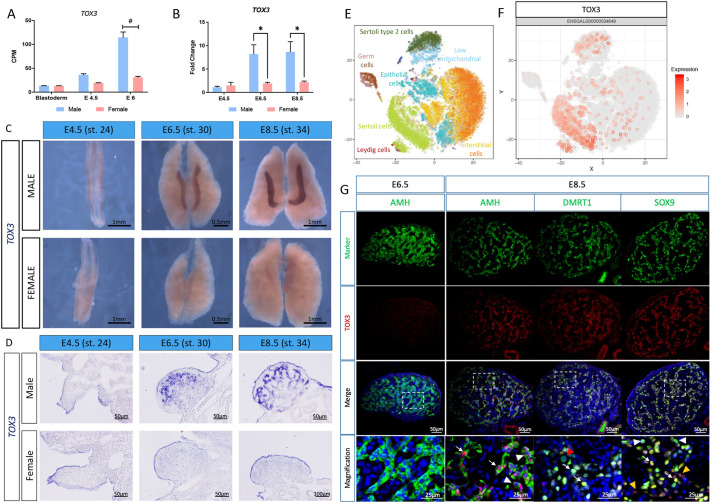
**TOX3 is upregulated in chicken supporting cells after the onset of sex differentiation.** (A) *TOX3* mRNA expression levels from bulk gonadal RNA-seq in count per million (CPM) at blastoderm stage, E4.5 and E6. ^#^false discovery rate (FDR) <0.001. (B) *TOX3* mRNA expression by qRT-PCR, relative to β-actin and normalized to an E4.5 male. Data are mean±s.e.m., *n*=6. *adjusted *P*-value<0.05. Multiple *t*-test and Holm-Sidak post-test. (C) *TOX3* mRNA expression by whole-mount *in situ* hybridization in the urogenital system. (D) Sections of the *TOX3* whole-mount *in situ* hybridization. (E) t-SNE plot of all gonadal male cells sequenced, color-coded by cell type. (F) Normalized expression of TOX3 on a t-SNE visualization of all male gonadal chicken cells. (G) E6.5 and E8.5 testicular immunofluorescence for Sertoli cell markers (AMH, DMRT1 and SOX9) and TOX3. White arrows indicate colocalization of TOX3 with the Sertoli cell markers. The areas outlined are shown at higher magnification below. White arrowheads indicate AMH-, SOX9- or DMRT1-positive, TOX3-negative cells. Yellow arrowheads indicate TOX3-positive SOX9-negative cells. Red arrowhead indicates a DMRT1-positive germ cells.

In the chicken embryo, the master testis gene *DMRT1* is expressed in the early male gonad (from at least E4.5/stage 25), before overt gonadal sex differentiation. Anti-Müllerian hormone (AMH) is expressed at low levels from the same time point, either directly or indirectly activated by DMRT1. The crucial testis factor SOX9 is first expressed later (at E6.0/stage 29) at the onset of testis cord organization (Estermann et al., 2021b). Gonadal TOX3 expression more closely resembles that of SOX9. Previous gonadal bulk RNA-seq performed in our laboratory showed that *TOX3* expression is sexually dimorphic at the onset of gonadal sex differentiation (embryonic day 6, E6/HH stage 29), being upregulated in male gonads (Fig. 1A) (Ayers et al., 2015; Hamburger and Hamilton, 1951). To validate these results, *TOX3* gonadal expression was quantified by qRT-PCR before (E4.5, stage 24), at the onset (E6.5, stage 30) and after (E8.5, stage 34) the onset of gonadal sex differentiation. *TOX3* was lowly expressed in both male and female gonads at E4.5 (Fig. 1B). *TOX3* mRNA expression was upregulated in developing male gonads at E6.5/stage 30 and E8.5/stage 34, whereas it remained low in female gonads (Fig. 1B). These data are consistent with the RNA-seq results. To define the spatial expression pattern of *TOX3* in embryonic gonads, whole-mount *in situ* hybridization (WISH) was performed on male and female urogenital systems at E4.5 (stage 24), E6.5 (stage 30) and E8.5 (stage 34). Results showed positive staining in male gonads, but not in females, after the onset of gonadal sex determination (E6.5), consistent with qRT-PCR data (Fig. 1C). WISH gonadal transverse sections showed that, in males, *TOX3* was expressed in the developing seminiferous cords of the gonadal medulla (Fig. 1D).

To identify the specific testicular cell types expressing *TOX3*, our previous chicken testis single-cell RNA-seq data were scrutinized (Estermann et al., 2020). A t-SNE containing E4.5, E6.5, E8.5 and E10.5 whole-testis samples was generated, identifying the main testicular cell populations (Fig. 1E). *TOX3* expression was mainly restricted to the Sertoli cell lineage (lime green), as well as in the Sertoli cells in the Sertoli-Leydig cell sub-cluster (Fig. 1F). *TOX3* was not expressed in the Leydig cells in that sub-cluster (Burgundy). To confirm this expression pattern, immunofluorescence was performed in E6.5 and E8.5 testicular sections. Although *TOX3* mRNA expression was detected at E6-E6.5, TOX3 protein was not detected at this stage, indicating a delay in TOX3 translation or possibly expression below the level of detection by the antibody (Fig. 1G). At E8.5, TOX3 protein showed nuclear localization, as expected of a transcription factor, and was detected inside the testicular cords, consistent with the mRNA expression. TOX3 was expressed in supporting cells, colocalizing with SOX9, AMH and DMRT1 (Fig. 1G, white arrows), but not in the DMRT1^+^ germ cells (Fig. 1G, red arrowhead). Some AMH^+^, SOX9^+^ supporting cells were negative for TOX3 (Fig. 1G, white arrowheads), suggesting that only a subset of supporting cells expresses TOX3, or that Sertoli cells are asynchronous in the timing of their expression of the protein. Interestingly, TOX3-positive AMH/SOX9-negative cells were also detected (Fig. 1G, yellow arrowheads).

### TOX3 knockdown results in increased Leydig cell differentiation

To evaluate the role of TOX3 on testicular differentiation, two different *TOX3* shRNAs (*sh370* and *sh685*) were cloned into the *RCASBP(A)* viral vector expressing a BFP reporter and the shRNA (Estermann et al., 2021a). To test the ability of the shRNA to knockdown *TOX3* expression, DF-1 cells (a chicken embryo fibroblast cell line) were transfected with RCASBP plasmids expressing one or other of these shRNAs. As a control, DF-1 cells were transfected with a non-silencing *shRNA* (Estermann et al., 2021a). After all cells had become BFP positive, they were transfected with a *RCASBP(D)-GFP-T2A-TOX3* overexpression plasmid. At 48 h post-transfection, cells were fixed and *TOX3* knockdown was assessed by reduction of GFP expression. *TOX3 sh685* showed the greatest reduction of GFP (and hence TOX3) expression (Fig. S1A). To quantify this reduction, flasks containing DF-1 cells were transfected with either *TOX3 sh685* or non-silencing shRNA. At 72 h post-transfection, DF-1 cells were collected and plated in a 24-well plate and were left resting for 24 hours. Subsequently, cells were transfected with an *RCASBP(D)-GFP-T2A-TOX3* overexpression plasmid and 48 h post-transfection they were collected for RNA extraction. *TOX3* expression was quantified by qRT-PCR, showing significant reduction (60%) of *TOX3* expression in cells expressing *TOX3 sh685* compared with the control (Fig. S1B).

**Fig. 2.**
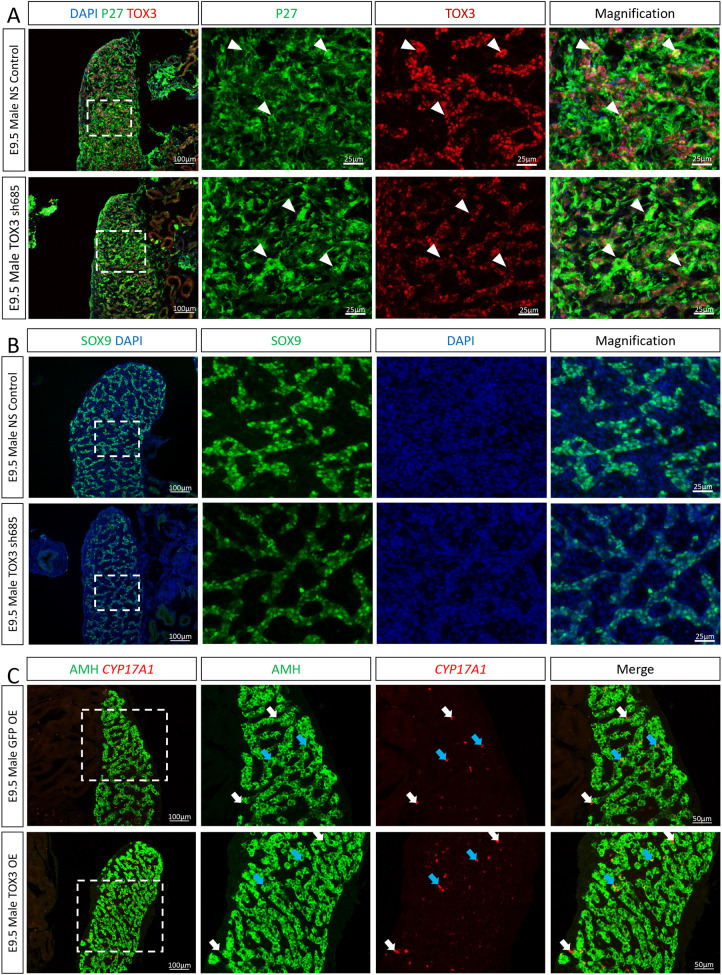
**TOX3 knockdown results in increased Leydig cell differentiation.** *TOX3 sh685* or non-silencing (NS) *shRNA* (control) plasmids were electroporated in chicken E2.5 coelomic epithelium. (A) Immunofluorescence detection of P27 (a marker showing robust viral vector delivery; green) and TOX3 (red) in E9.5 male gonadal sections. White arrowheads indicate shRNA-expressing supporting cells (*n*=6). (B) SOX9 immunofluorescence in control or TOX3 knockdown E9.5 male gonads (*n*=6). (C) *CYP17A1* fluorescent *in situ* hybridization and AMH (Sertoli cell marker) immunofluorescence in TOX3 knockdown or control E9.5 male gonads (*n*=4). White arrows indicate *CYP17A1*-positive AMH-negative Leydig cells. Blue arrows indicate intermediate cells (*CYP17A1* and *AMH* positive). The areas outlined are shown at higher magnification on the right.

To evaluate the effect of knocking down *TOX3 in vivo*, RCASBP(A)TOX3-Sh685 virus or non-silencing control were injected into embryos at the blastoderm stage (day 0 of incubation). All the TOX3 knockdown embryos died at early stages of development, suggesting embryonic lethality when TOX3 knockdown occurred globally. To overcome this problem, *RCASBP(A)TOX3-Sh685* or non-silencing control plasmids were electroporated into the left coelomic epithelium of E2.5 chicken embryos. This afforded a more-targeted delivery. Urogenital systems were harvested at E9.5 (stage 35/36) (Fig. S1C), and immunofluorescence was performed on male (ZZ) gonads. As electroporation is innately variable, even across one gonad, we rely on localized immunofluorescence intensity as a measure of knockdown. Although not quantitative, it is clear from Fig. 2A that endogenous gonadal expression of TOX3 is lower after treatment with the specific shRNA. In the non-silencing control, TOX3 expression was uniform along the testicular cords, colocalizing with GFP (Fig. 2A). SOX9 expression was also reduced upon TOX3 knock down (Fig. 2B), whereas AMH or DMRT1 expression remained unchanged (Fig. S2A,B).

We have previously reported that embryonic steroidogenic cells in the chicken embryo derive from a subset of previously committed supporting cells, in both males and females (Estermann et al., 2020). This process of differentiation involves a sequential upregulation of the diagnostic steroidogenic cell marker *CYP17A1* and a subsequent downregulation of the supporting cell markers (Estermann et al., 2020). Our single-cell data show that *TOX3* was not only expressed in the supporting cells, but also in a subpopulation of Sertoli cells that clusters transcriptionally with steroidogenic Leydig cells (Fig. S2C). Interestingly, *TOX3* is not expressed in the cells with the highest expression of *CYP17A1* (Fig. S2D), suggesting that *TOX3* might have a role in the Leydig cell differentiation. To address the role of *TOX3* in fetal Leydig cell differentiation, RCASBP(A)TOX3-Sh685 or non-silencing control plasmids were electroporated in the coelomic epithelium of E2.5 chicken embryos. Urogenital systems were harvested at E9.5, and *CYP17A1* fluorescence *in situ* hybridization was conducted to detect steroidogenic cells, followed by immunofluorescence against the Sertoli cell marker AMH (Fig. 2C). *TOX3* knockdown resulted in an increased number of *CYP17A1*^+^ Leydig cells, compared with the non-silencing control (Fig. 2C). These data suggest that TOX3 is required to inhibit Leydig cell differentiation.

### TOX3 overexpression in male gonads inhibits Leydig cell differentiation

To evaluate the effect of TOX3 in gonadal differentiation, the *TOX3* open reading frame was cloned into RCASBP(D) viral vector [coupled to GFP reporter, as *RCASBP(D)-GFP-T2A-TOX3*]. DF-1 cells were transfected with this construct, and TOX3 protein and GFP expression were detected by immunofluorescence (Fig. S3A). Both nuclear TOX3 and cytoplasmic GFP were co-expressed in the transfected cells, validating the overexpression construct (Fig. S3A). Quantitative RT-PCR was also used to confirm *TOX3* mRNA overexpression in DF-1 cells, showing a significant increased expression compared with the control (a vector expressing only GFP) (Fig. S3B).

**Fig. 3.**
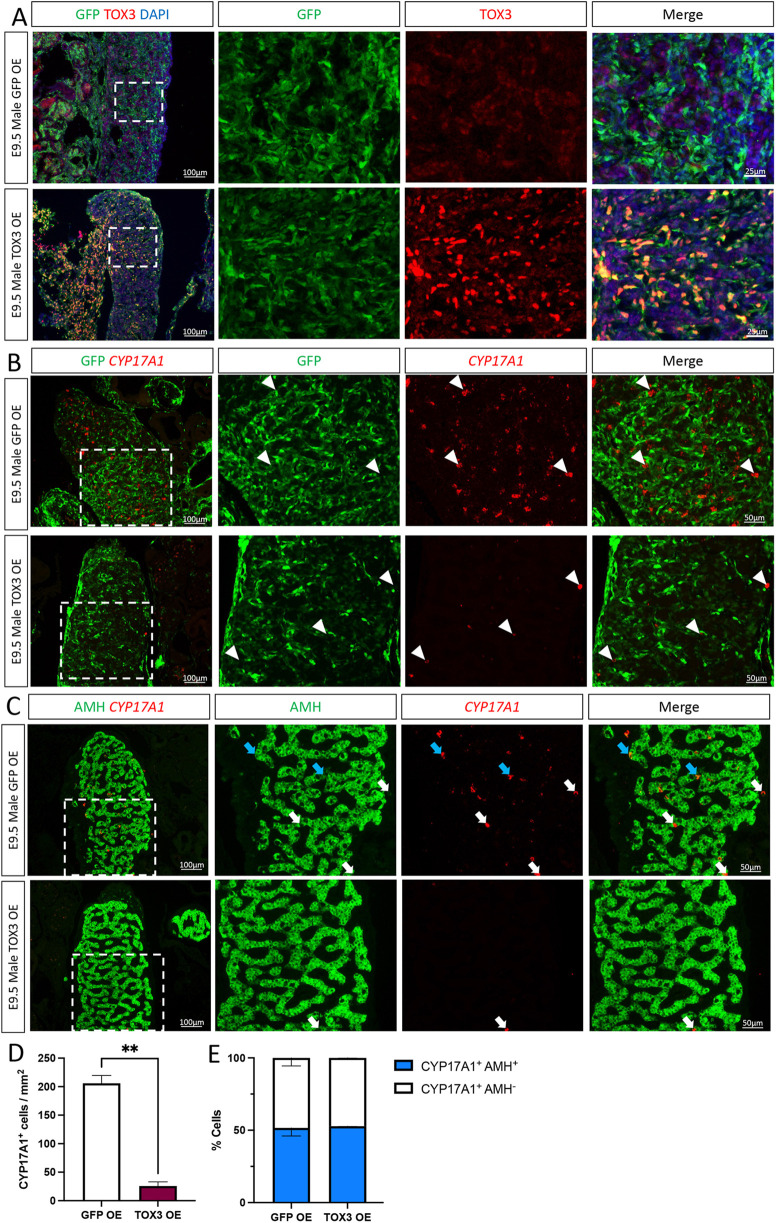
**TOX3 overexpression in male gonads inhibits Leydig cell differentiation.** TOX3 or GFP (control) overexpression plasmids were electroporated in chicken E2.5 coelomic epithelium. Male gonads were examined at E9.5. (A) Immunofluorescence was performed to detect GFP and TOX3 (*n*=6). (B) CYP17A1 fluorescence *in situ* hybridization was performed, followed by GFP immunofluorescence (*n*=3). White arrowheads indicate steroidogenic (CYP17A1-positive) cells. (C) CYP17A1 fluorescence *in situ* hybridization was performed, followed by AMH immunofluorescence (*n*=3). The areas outlined are shown at higher magnification on the right. White arrows indicate CYP17A1-positive AMH-negative Leydig cells. Blue arrows indicate intermediate cells (CYP17A1 and AMH positive). (D) Quantification of CYP17A1-positive cells per gonadal area in control [GFP overexpressing (OE)] or TOX3 OE testis. Data are mean±s.e.m., *n*=3, **P<0.01 (unpaired two-tailed *t*-test). (E) Proportion of steroidogenic (CYP17A1^+^ AMH^−^) and intermediate (CYP17A1^+^ AMH^+^) cells in control (GFP OE) or TOX3 OE testis. Data are mean±s.e.m., *n*=3. Two-way ANOVA, Tukey's post-hoc test.

To address the role of *TOX3* in gonadal development *in vivo*, TOX3 was overexpressed in embryonic chicken gonads by coelomic electroporation at E2.5 using the RCASBP vector described previously. As a control, a RCASBP plasmid expressing GFP was electroporated. TOX3 was successfully overexpressed in E9.5 male gonads, colocalizing with GFP expression in the developing testis (Fig. 3A). No changes in Sertoli cell markers AMH, SOX9 and DMRT1 were detected when TOX3 was overexpressed in male gonads (Fig. S4A-C). Additionally, the pre-granulosa marker aromatase was not detected in male gonads upon TOX3 overexpression (Fig. S4D). This suggests that TOX3 overexpression in male gonads has no effect in Sertoli cell differentiation.

To evaluate the role of TOX3 overexpression in Leydig cell differentiation*, CYP17A1* fluorescent *in situ* hybridization was performed (Fig. 3B,C). Male gonads overexpressing *TOX3* showed 87% fewer *CYP17A1*-positive cells than the control (Fig. 3B,D). Consistent with our previous finding that steroidogenic cells arise from a sub-population of AMH^+^ supporting (Sertoli) cells, CYP17A1^+^ cells were located both within the developing testis cords and outside them. Interestingly, the ratios of ‘intermediate cells’ (expressing both CYP17A1 and AMH) and Leydig cells (CYP17A1^+^ but AMH^−^) remained unaffected after TOX3 knockdown (Fig. 3C,E). Taken together, these data suggest that *TOX3* has a role in maintaining the identity of supporting cells by inhibiting the differentiation of steroidogenic Leydig cells and the induction of *CYP17A1* expression.

### TOX3 misexpression in female gonads inhibits ovarian steroidogenic cell differentiation

**Fig. 4.**
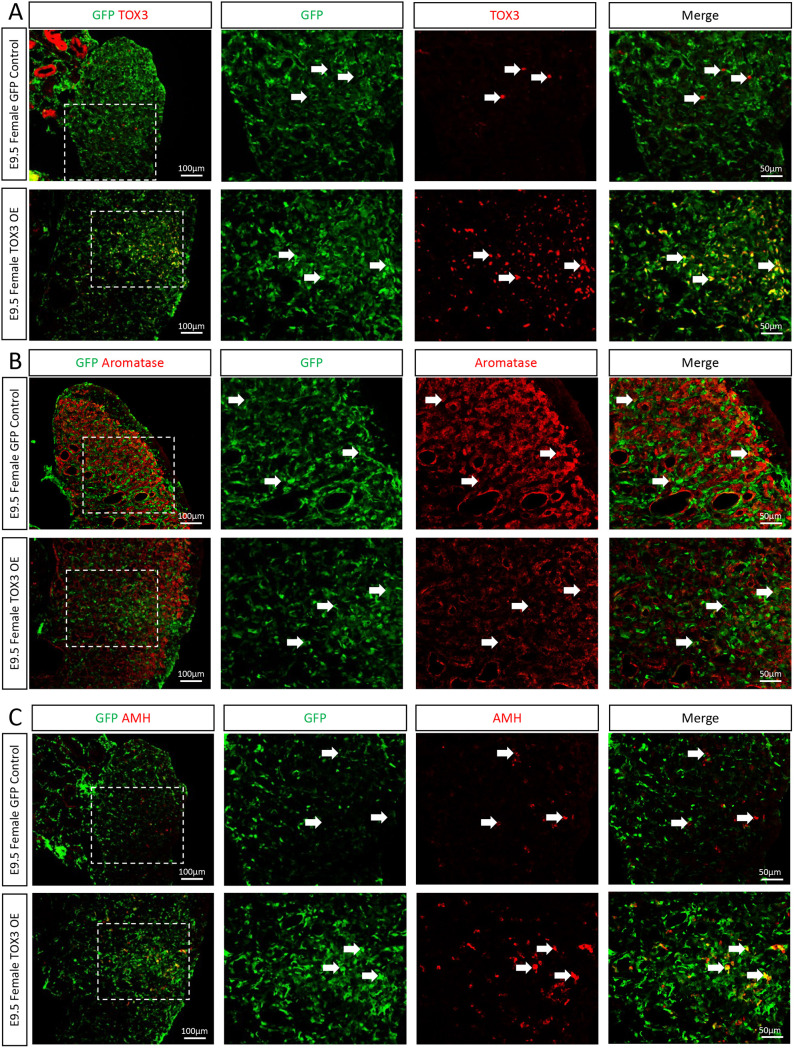
**TOX3 overexpression in ovaries downregulates aromatase.** (A-C) TOX3 or GFP (control) overexpression plasmids were electroporated in chicken E2.5 coelomic epithelium (*n*=5). Female gonads were collected at E9.5 and immunofluorescence was performed to detect GFP and (A) TOX3, (B) aromatase or (C) AMH. The areas outlined are shown at higher magnification on the right. White arrows indicate GFP-positive cells.

To evaluate the effect of ectopic expression of TOX3 in the developing ovary and, in particular, in supporting and steroidogenic cell differentiation, the TOX3 overexpression construct was electroporated in the left ovary. *TOX3* was successfully misexpressed in female gonads, colocalizing with GFP reporter expression in the developing ovary (Fig. 4A). Female gonads overexpressing TOX3 showed a reduction in aromatase expression in the region of the gonad misexpressing TOX3, compared with the GFP control (Fig. 4B). The cortical (non-medullary) region of the gonads was also affected, showing a thinner cortical structure in TOX3 misexpressing ovaries (Fig. S5A). As cortical development is sensitive to estrogens (Guioli et al., 2020), this could be a secondary effect of aromatase downregulation. Despite reduction in the cortical domain in TOX3-expressing gonads, germ cells exhibited a normal localization in the cortical region (Fig. S5B). Additionally, TOX3 overexpressing ovaries showed an increased expression of the male marker AMH (colocalizing with GFP), compared with the control (Fig. 4C). No SOX9 or DMRT1 upregulation was detected (Fig. S5C,D). In fact, DMRT1 appeared to be downregulated in female cells expressing TOX3 in the medulla (Fig. S5D).

**Fig. 5.**
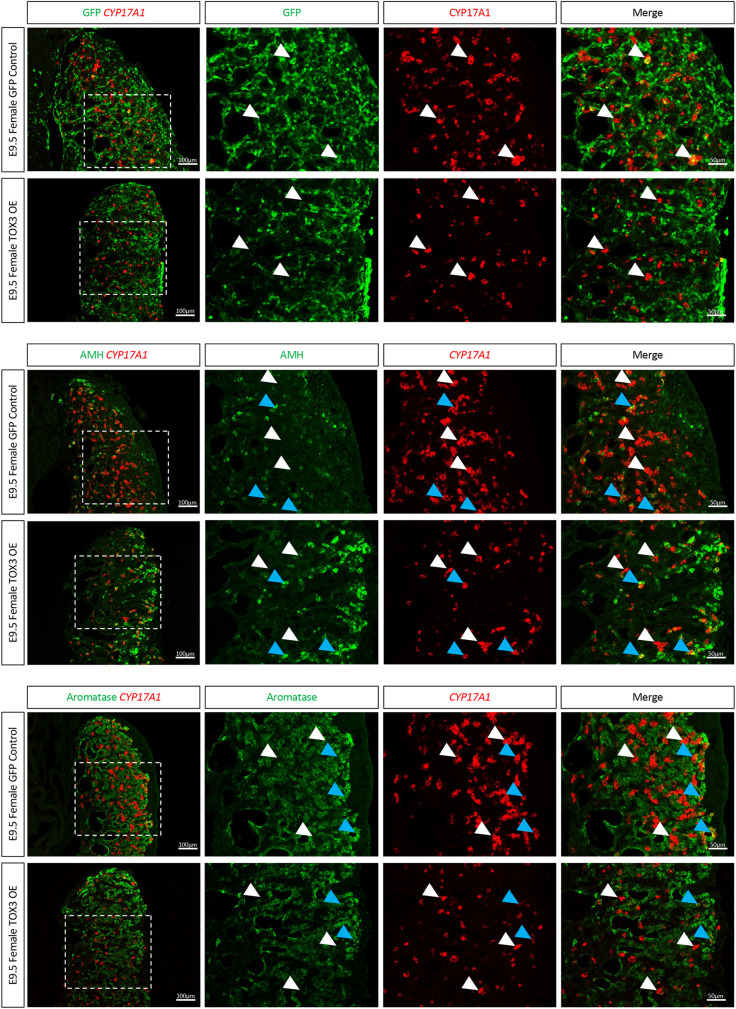
**TOX3 overexpression inhibits steroidogenic theca cell differentiation.** TOX3 or GFP (control) overexpression plasmids were electroporated in chicken E2.5 coelomic epithelium (*n*=3). Female gonads were collected at E9.5 and CYP17A1 fluorescence *in situ* hybridization was performed, followed by (A) GFP, (B) AMH or (C) aromatase immunofluorescence. White arrowheads indicate steroidogenic (CYP17A1-positive) cells. Blue arrowheads indicate intermediate (double-positive) cells. The areas outlined are shown at higher magnification on the right.

As chicken embryonic theca and Leydig cells share a similar transcriptome, with no evident sex-specific markers (Estermann et al., 2020), we tested whether TOX3 misexpression in females could modulate steroidogenic cell differentiation. Ovarian TOX3 misexpression resulted in a reduction in the population of steroidogenic *CYP17A1*-positive theca cells (Fig. 5A-C). In TOX3 misexpressing gonads, GFP and CYP17A1 did not colocalize. This suggests that the remaining *CYP17A1*-positive cells were TOX3 (GFP) negative (Fig. 5A). Additionally, TOX3 misexpression resulted in AMH upregulation in females (Fig. 5B), concomitant with aromatase downregulation (Fig. 5C), consistent with the previous results. Similar to TOX3 overexpression in males, intermediate cells were also present in the gonad, co-expressing CYP17A1 and AMH (Fig. 5B) or aromatase (Fig. 5C). Taken together, the data suggest that, in females, TOX3 protein can inhibit estrogenic (aromatase^+^) and androgenic (*CYP17A1*^+^) cell differentiation.

### Estrogen inhibits TOX3 expression

**Fig. 6.**
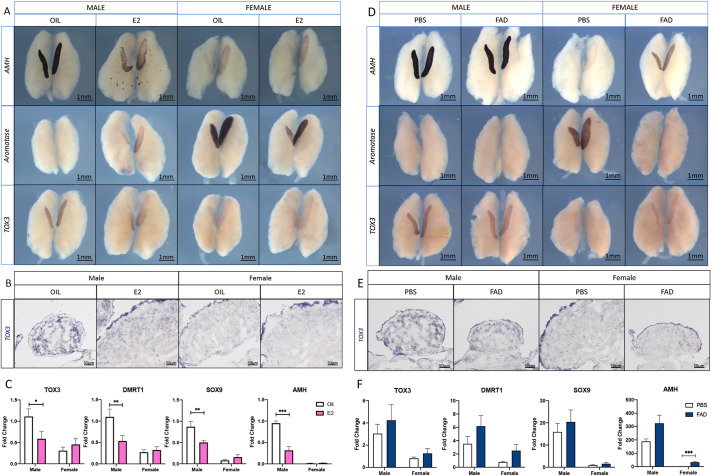
**Estrogen inhibits TOX3 expression.** (A-C) 17β-estradiol (E2) or vehicle (sesame oil) was injected into E3.5 chicken eggs. Male and female gonads were collected at E9.5 for TOX3 whole-mount *in situ* hybridization (A,B) or for qRT-PCR (C). (A) Downregulation of *AMH*, upregulation of aromatase and downregulation of *TOX3* in male gonads exposed to E2 compared with sesame oil control (*n*=6). (B) *TOX3* whole-mount *in situ* hybridization overstained sections (*n*=6). (C) *TOX3*, *DMRT1*, *SOX9* and *AMH* mRNA expression levels by qRT-PCR. Expression is relative to β-actin and normalized to male oil samples. (D-F) Fadrozole (FAD) or vehicle control (PBS) was injected into E3.5 chicken eggs. Male and female gonads were collected at E9.5 for TOX3 whole-mount *in situ* hybridization (D,E) or qRT-PCR (F). (D) Upregulation of AMH, downregulation of aromatase but no effect upon TOX3 mRNA expression in female gonads exposed to FFAD compared with PBS control (*n*=3). (E) TOX3 whole-mount *in situ* hybridization overstained sections (*n*=3). (F) qRT-PCR for *TOX3*, *DMRT1*, *SOX9* and *AMH*. Expression is relative to β-actin and normalized to female PBS samples (*n*=6). Data are mean±s.e.m. *P<0.05, **P<0.01 and ***P<0.001 (multiple *t*-test and Holm-Sidak post-hoc test).

Steroid hormones influence avian gonadal sex differentiation. Specifically, estrogen is required for ovarian differentiation (Scheib, 1983). The estrogen-synthesizing enzyme, aromatase, is activated only in female gonads at the onset of gonadal sex differentiation, and the estrogen that it produces has a positive feedback effect upon further aromatase gene transcription. Estrogen synthesized in the gonadal medulla regulates the adjacent cortex, inducing proliferation (Guioli et al., 2020). Conversely, exposure of the male embryo to exogenous estrogens can feminize gonads (Guioli et al., 2020; Major and Smith, 2016; Shioda et al., 2021; Vaillant et al., 2001a; Hirst et al., 2017). To understand the regulation of *TOX3* expression, 17β-estradiol (E2) or vehicle (sesame oil) were injected into chicken eggs at E3.5, before the onset of gonadal sex differentiation and male upregulation of TOX3. E9.5 urogenital systems were then collected and processed for aromatase, *AMH* and *TOX3* whole-mount *in situ* hybridization (Fig. 6A,B) or for qRT-PCR (Fig. 6C).

Male left gonads exposed to estrogens were larger than the controls, showing a female-like left-right asymmetry and suggesting male-to-female sex reversal. *AMH* and *TOX3* expression were reduced in male gonads exposed to 17β-estradiol (E2) (Fig. 6A), whereas expression of the normally female-restricted marker, aromatase, was induced in males (Fig. 6A). Gonadal sections of *TOX3* WISH revealed that male left gonads exposed to E2 had an ovarian-like structure that lacked *TOX3* expression in the medulla (Fig. 6B). *TOX3* expression levels were quantified by qRT-PCR, showing a significant reduction of its expression in male gonads exposed to 17β-estradiol (E2), compared with the control (sesame oil) (Fig. 6C). Additionally, the Sertoli cell markers *SOX9*, *DMRT1* and *AMH* also showed a significant reduction in expression (Fig. 6C).

Inhibiting aromatase function with the drug fadrozole induces masculinization of female gonads (Vaillant et al., 2001a,b; Elbrecht and Smith, 1992). When female embryos were treated with fadrozole, *TOX3* was not upregulated in the gonad (Fig. 6D-F). This was despite the downregulation of aromatase (Fig. 6D). Furthermore, *AMH* but not *SOX9* or *DMRT1* was significantly upregulated in feminization experiments (Fig. 6D,F). Taken together, these data suggest that *TOX3* expression is negatively regulated by estrogens, directly or indirectly. However, the absence of estrogens is not sufficient to induce *TOX3* expression, pointing to the requirement of a male-specific factor.

### DMRT1 regulates *TOX3* expression

**Fig. 7.**
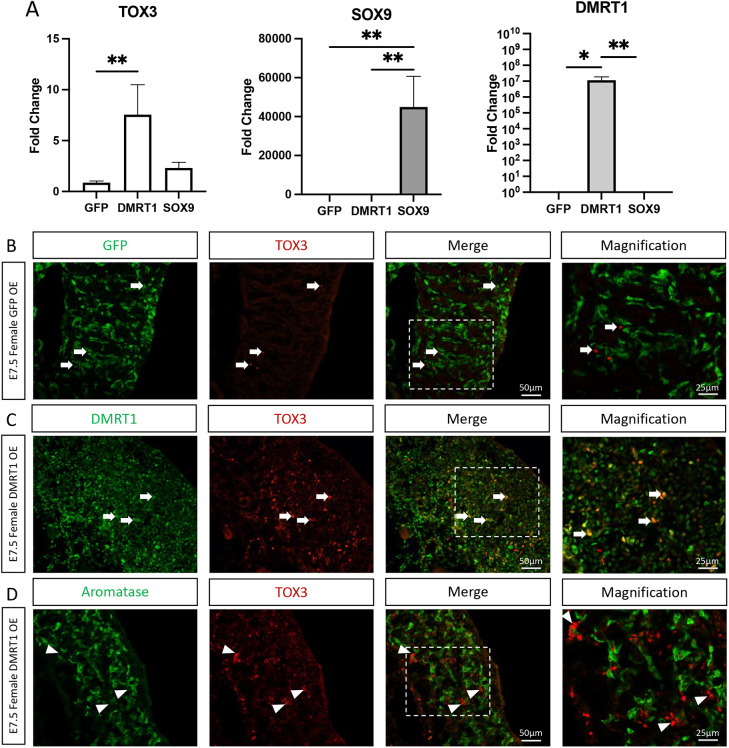
**DMRT1 overexpression induces TOX3 expression *in vitro* and *in vivo*.** (A) DMRT1, SOX9 or GFP as a control were overexpressed *in vitro* in DF-1 chicken fibroblastic cells, and *TOX3*, *SOX9* and *DMRT1* mRNA expression was measured by qRT-PCR. Expression is relative to β-actin and normalized to GFP overexpression control. Data are mean±s.e.m. *P<0.05 and **P<0.01 (multiple *t*-test and Holm-Sidak post-hoc test) (*n*=6). (B-D) TOX3 and GFP (B), DMRT1 (C) or aromatase (D) immunofluorescence in E7.5 female gonadal sections overexpressing GFP (control) or DMRT1 (*n*=4). The areas outlined are shown at higher magnification on the right. White arrows indicate TOX3-positive cells. White arrowheads indicate TOX3-positive aromatase-negative cells.

The Z-linked transcription factor DMRT1 is the master testis-determinant in chicken. *DMRT1* knockout or knockdown induces feminization or complete ovary formation (Smith et al., 2009a; Hirst et al., 2017; Ioannidis et al., 2021). Targeted overexpression of *DMRT1* alone induces upregulation of *SOX9* and *AMH* (male-specific genes) in female chicken gonads (Lambeth et al., 2014). In mammals, the main role of the sex-determining gene *Sry* is to induce the expression of *Sox9*, which promotes testicular differentiation by activating pro-testis genes and repressing pro-ovarian genes (Hiramatsu et al., 2009; Li et al., 2014; Wilhelm et al., 2007; Qin and Bishop, 2005). To understand how *TOX3* is regulated in the embryonic chicken testis, DMRT1, SOX9 or GFP (as a control) were overexpressed in DF-1 cells, a chicken fibroblastic cell line. *DMRT1*, *SOX9* and *TOX3* mRNA expression levels were evaluated by qRT-PCR. DMRT1 overexpression, but not SOX9, was able to significantly upregulate *TOX3* expression *in vitro* (Fig. 7A).

**Fig. 8.**
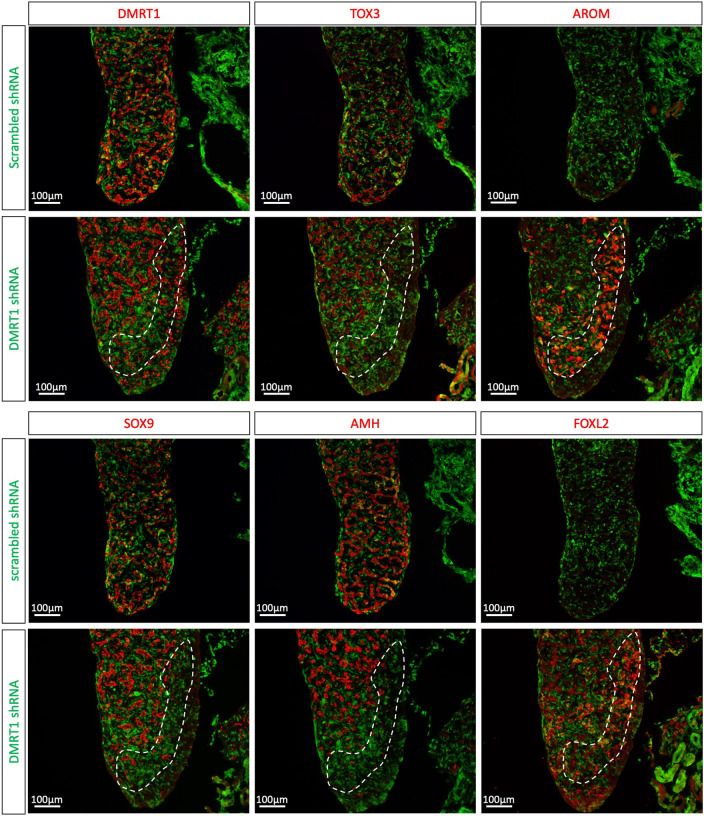
***In vivo* DMRT1 knockdown inhibits TOX3 expression in male gonads.** RCAS virus expressing *DMRT1 shRNA343* or scrambled shRNA were injected at the blastoderm stage. Gonads were examined at E9.5 (*n*=4) and immunofluorescence against GFP (transfection marker), DMRT1, TOX3, aromatase (AROM), SOX9, AMH and FOXL2 was performed. The area outlined indicates a DMRT1 knockdown gonadal region in supporting cells.

To evaluate whether it was sufficient to induce *TOX3* expression *in vivo*, *DMRT1* was overexpressed in chicken gonads by coelomic electroporation at E2.5, as reported previously (Lambeth et al., 2014). As a control, a GFP-expressing plasmid was electroporated. Immunofluorescence for TOX3 was performed on E7.5 female gonads. In female control gonads, TOX3 protein expression was minimal (Fig. 7B). In contrast, when DMRT1 was misexpressed in female gonads, TOX3 was upregulated, colocalizing with DMRT1 (Fig. 7C). In addition, TOX3-positive cells did not colocalize with the pre-granulosa marker aromatase, suggesting that TOX3 is in fact expressed in DMRT1-induced Sertoli cells (Fig. 7D). To evaluate whether it is necessary to regulate *TOX3* expression, *DMRT1* was knocked down by viral injection, using the blastoderm delivery of a shRNA method reported previously (Smith et al., 2009a). In these experiments, a GFP reporter was used, marking those cells infected with virus expressing the DMRT1-specific shRNA or the control (scrambled shRNA). The *DMRT1* shRNA has been previously validated and published by our laboratory, showing robust knockdown of *DMRT1* mRNA and protein expression (Smith et al., 2009a). Control E9.5 male gonads expressing the non-silencing control shRNA (scrambled shRNA) showed normal DMRT1, SOX9, AMH and TOX3 expression in the testicular cords, and no expression of female pre-granulosa markers (FOXL2 and aromatase) (Fig. 8). In contrast, aromatase and FOXL2 were locally upregulated in male gonads in regions where DMRT1 expression was knocked down, colocalizing with the GFP reporter (and hence shRNA delivery) (Fig. 8). GFP-positive cells were TOX3 negative, indicating that in the cells where DMRT1 was downregulated, TOX3 expression was not expressed or was lost (Fig. 8). Other Sertoli markers, SOX9 and AMH, were also absent in the DMRT1 knockdown region of the gonad, where there is no colocalization with GFP (Fig. 8), consistent with our previous data (Smith et al., 2009a). Taken together, these data indicate that DMRT1 is necessary and sufficient to induce *TOX3* expression.

## DISCUSSION

**Fig. 9.**
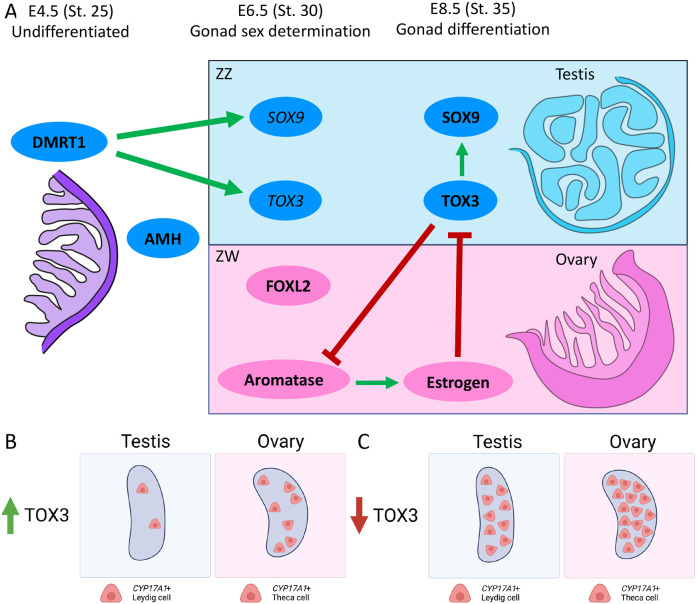
**The role of TOX3 in gonadal differentiation.** (A) DMRT1 and AMH are expressed in the undifferentiated gonads. DMRT1, the sex determining gene, regulates the mRNA expression of *SOX9* and *TOX3* in ZZ males at E6.5. TOX3 and SOX9 protein expression is detected later in development. In this study, we show that TOX3 is required to regulate SOX9 expression. Additionally, TOX3 inhibits aromatase expression when overexpressed in female (ZW) gonads. FOXL2 and aromatase upregulation denotes the beginning of ovarian sex differentiation. Estrogens synthesized by the enzyme aromatase inhibit *TOX3* expression in the ovary. (B) High levels of TOX3 inhibit steroidogenic cell differentiation in both male and female gonads. (C) By contrast, low levels of TOX3, as in the normal ovary or in TOX3 knockdown experiments in male gonads, results in a high number of steroidogenic cells.

We first identified *TOX3* as a previously unreported gene expressed in pre-Sertoli cells based on a single-cell RNA-seq screen (Estermann et al., 2020). *TOX3* mRNA was found to be upregulated in male but not female gonads after the onset of gonadal sex differentiation (between E6.5/stage 30 and E8.5/stage 35) (Fig. 9A). TOX3 protein was not detected at E6.5, suggesting that there is a translational delay or that the levels of protein expression were not high enough to be detected by immunostaining (Fig. 9A). Additionally, TOX3 protein expression was not homogeneous in the testicular cords, colocalizing partially with AMH and SOX9. This could reflect expression in Sertoli cells at different (asynchronous) stages of development or could reflect expression in a distinct subset of cells. These findings agree with the chicken testicular single-cell RNA-seq where two different Sertoli cell types were identified, suggesting that the Sertoli cell population is not as homogeneous as generally considered (Estermann et al., 2020). A similar phenomenon has also been described for the chicken Z-linked gene *HEMGN*, which exhibits variable levels of expression in the nuclei of developing male gonads (Lambeth et al., 2014). *HEMGN* encodes a transcription factor that is expressed in developing chicken Sertoli cells and required for testis development (Nakata et al., 2013). It lies downstream of DMRT1 in the male developmental pathway in chicken, although it might not be conserved in other birds (Hirst et al., 2017). It would be of interest to determine whether the variable TOX3 expression spatially coincides with the HEMGN^+^ Sertoli cells.

The data presented here indicate that DMRT1 activates *TOX3* during testicular development in the chicken embryo (Fig. 9A). *DMRT1* knockdown in male gonads results in localized loss of TOX3 protein expression, while DMRT1 misexpression in females causes upregulation of TOX3 expression. *TOX3* is likely to be one of many genes activated by DMRT1, generating a network of factors that coordinate testis formation and function. We and others have previously shown that DMRT1 directly or indirectly regulates *SOX9* and *AMH* during chicken testis development (Lambeth et al., 2014; Ioannidis et al., 2021). Knockdown of *TOX3* expression resulted in a lower level of SOX9 in male gonads, but testis cords still formed and AMH was still expressed. This suggests that TOX3 might participate in regulating SOX9 expression (Fig. 9A). But TOX3 is not the only factor regulating SOX9, as it fails to induce SOX9 expression in the overexpression experiments. This could be explained by the lack of DMRT1 expression, suggesting that TOX3 and DMRT1 (and may be other factors) are required to induce SOX9 expression.

Misexpression of TOX3 in female gonads had an effect upon the endocrine development of the gonad, by locally activating AMH and suppressing aromatase expression (Fig. 9A). As *TOX3* knockdown in males did not affect AMH expression, it is likely that ectopic TOX3 in the female gonad affects AMH expression indirectly by suppressing aromatase expression. Estrogen and AMH are mutually antagonistic in the avian gonad (Lambeth et al., 2013, 2016; Vaillant et al., 2001b). Suppression of aromatase, and hence estrogen synthesis, allows upregulation of *AMH* expression in the female gonad (Fig. 6D) (Nishikimi et al., 2000). Conversely, exogenous estrogen (E2, 17-β-estradiol) causes AMH mRNA downregulation in male gonads (Fig. 6A). Hence, here, TOX3 may have led to increased AMH in the female chicken gonad via local suppression of aromatase. In the male gonad, one of the roles of TOX3 in the supporting cell lineage (Sertoli cells) may be to ensure that the aromatase gene is inactive, hence preventing feminization (Fig. 9A). It would be of interest to scrutinize the *CYP19A1* gene, which encodes aromatase, for TOX3-binding sites. Conversely, *TOX3* was downregulated in male gonads exposed to exogenous estrogen, suggesting a mutually antagonistic regulation (Fig. 9A).

In the female avian embryo, ovarian cortex development is dependent on estrogens, which are synthesized in the medullary cord cells (pre-granulosa type cells). Accordingly, ERα is expressed in the gonadal cortex (Lambeth et al., 2013; Guioli et al., 2020). When *TOX3* was overexpressed in female gonads, the cortical area was reduced. This could also be a secondary effect of the reduction in expression of aromatase, the enzyme responsible for transforming androgens into estrogens. Additionally, TOX3 misexpression inhibited steroidogenic embryonic theca cell differentiation (Fig. 5). Fewer embryonic theca cells may cause a reduction in androgens and, as androgens are the substrate for estrogen synthesis, in lower levels of estrogens and thus a smaller cortex. Surprisingly, despite having a thinner cortex, germ cells remained in the cortical or juxtacortical region and not in the medulla when TOX3 was misexpressed in females. This could suggest that, in fact, the germ cells are not surrounded by epithelial cells but by so-called juxtacortical medulla cells. The juxtacortical medulla is compact region resulting from the accumulation of mesenchymal cells directly underneath the chicken ovarian cortex (Estermann et al., 2021a; Oreal et al., 2002). This ovarian structure is poorly understood but it is thought to regulate germ cell meiosis, as it expresses the retinoic acid degradation enzyme *CYP26B1* (Smith et al., 2008a). If the germ cells are located in the juxtacortical medulla, it could explain previous reports of the presence of FOXL2-positive cells, presumably pre-granulosa cells, in the juxtacortical medullary region of E14.5 chicken ovaries (Major et al., 2019).

The data presented here indicate that another role of *TOX3* in the developing chicken gonad is modulation of steroidogenic cell differentiation. Overexpression in both male and female gonads reduced the pool of *CYP17A1*^+^ steroidogenic cells (Fig. 9B), whereas knockdown in males resulted in an increased number of *CYP17A1*^+^ cells (Fig. 9C). In the embryonic chicken testis, we have shown that the *CYP17A1*^+^ pre-Leydig cell progenitors likely derive from a subset of the Sertoli cells (Estermann et al., 2020). When TOX3 overexpression was performed, there was a clear loss of *CYP17A1* expression, presumably reflecting a loss of steroidogenic progenitor cells. This suggests that TOX3 may normally act to restrain or modulate the sub-population of Sertoli cells that differentiates into the *CYP17A1*^+^ embryonic steroidogenic Leydig cells around the seminiferous cords. During this Sertoli-to-Leydig cell differentiation process, a subset of AMH-positive cells upregulates steroidogenic markers, such as *CYP17A1* (Estermann et al., 2020)*.* We consider these to be the transitioning population (Sertoli-to-Leydig cells). It is unknown whether all the Sertoli cells have the ability to differentiate into steroidogenic cells or whether specific factors are required to control this process. *TOX3* appears to be a key factor in this cell fate decision. As noted, not all AMH-positive pre-Sertoli cells were also TOX3 positive. Additionally, TOX3 overexpression in male gonads resulted in a reduction of both steroidogenic cells (*CYP17A1^+^*) and the intermediate/transitioning cells (AMH^+^/CYP17A1^+^). This suggests that *TOX3* inhibits or modulates the Sertoli-to-Leydig cell transition and maintains Sertoli cell identity. Based on this concept, TOX3^−^/AMH^+^ cells may differentiate into Leydig cells, whereas TOX3^+^/AMH^+^ cells may follow the Sertoli cell differentiation path. Further experiments involving lineage tracing and fate mapping are required to validate this theory. It is interesting, in this regard, that TOX3 downregulation has been reported to facilitate epithelial-to-mesenchymal transition by repression of *SNAI1* and *SNAI2* in cancer cells (Jiang et al., 2019). This would be consistent with our findings, as TOX3 overexpression appears to suppress the proposed transition of Sertoli (epithelial) to Leydig (mesenchymal) cells, when based on *CYP17A1* expression.

Alternatively, TOX3 expression in the supporting cell lineage may act indirectly by inducing paracrine factors that are secreted to regulate differentiation of the steroidogenic Leydig lineage. However, there are some caveats to these interpretations. First, the expression of *CYP17A1* is taken as a marker of the steroidogenic population. Loss of *CYP17A1* mRNA expression could reflect fewer pre-Leydig cells developing, as interpreted here, or it could simply reflect downregulation of that gene in the Leydig population. It would be of interest to examine *CYP17A1* expression together with cell proliferation markers and other markers of the steroidogenic lineage, such as *CYP11A1*, to distinguish these possibilities. In the mouse, 3β-HSD is widely used as a diagnostic marker of the male steroidogenic (fetal Leydig cell) population. However, in chicken, 3β-HSD is expressed in the embryonic Sertoli cells, not in the Leydig cell progenitors, and is therefore not an appropriate marker for the latter (Lambeth et al., 2016).

Human DNA variants in the *TOX3* locus have been associated with polycystic ovarian syndrome (PCOS) in several populations (Tian et al., 2020; Bakhashab and Ahmed, 2019; Pau et al., 2017; Liu et al., 2020). These mutational variants are not found in the coding region of *TOX3*, indicating that *TOX3* transcriptional regulation might be affected (Cui et al., 2014). PCOS is one of the most common infertility causes in females (Deswal et al., 2020). Hyperandrogenism (elevated androgen) is one of the main diagnostical characteristics of this syndrome (Rotterdam ESHRE/ASRM-Sponsored PCOS consensus workshop group, 2004). In adult PCOS granulosa cells, lower levels of TOX3 protein are detected, compared with the control (Ning et al., 2017). This suggests that lower levels of ovarian *TOX3* expression might act as causative factor for PCOS. The exact functional mechanism of *TOX3* in this disease or in the ovarian context is not known (Tian et al., 2020). The data presented here demonstrate that *TOX3* inhibits the differentiation of steroidogenic cells in embryonic chicken gonads. In PCOS, lower levels of *TOX3* could be implicated in an increase in steroid-producing cells, explaining the higher levels of androgens described in PCOS ovaries (Patel, 2018). This is consistent with our *TOX3* knockdown data (Fig. 2C). Additionally, rats with global lower levels for *Tox3* display obesity, sterility (male and female) and increased anxiety, all of which are symptoms of PCOS (Shunkwiler et al., 2018; Patel, 2018). Taken together, this indicates that downregulation of *TOX3* in ovaries could lead to PCOS in females by a dysregulation of steroidogenic cell differentiation. It has been suggested that dysregulated or abnormal embryonic gonadal development could be the cause of PCOS (Hartanti et al., 2020). Several PCOS-associated genes are expressed at different key developmental stages and in different cell types during ovarian development (Hartanti et al., 2020). In contrast, the expression levels of PCOS-associated genes have not been studied in development of the testicle, an organ specialized in androgen production. The current study described the role of a testicle-associated gene in polycystic ovarian syndrome, suggesting that abnormal embryonic gonadal sex differentiation could be one of the causes of PCOS. Additionally, further research is required to assess the expression levels and cell types where *TOX3* is expressed in adult gonads.

The data presented describe the role of *TOX3* in potentially maintaining Sertoli cell identity and inhibiting the steroidogenic lineage in embryonic chicken gonads. However, the mechanism is unclear. Omics technologies will be crucial to uncover *TOX3* function in gonadal differentiation. *In ovo* overexpression and knockdown experiments can be coupled with RNA-sequencing to identify genes regulated by *TOX3*. Additionally, TOX3 ChIP-seq will determine the direct target genes that TOX3 regulates. Furthermore, comparative analysis with other organisms is required to fully understand the roles of *TOX3* in gonadal development and, potentially, PCOS progression.

## MATERIALS AND METHODS

### Eggs and sexing

HyLine Brown fertilized chicken eggs (*Gallus domesticus*) were obtained from Research Poultry farm (Victoria, Australia) and incubated at 37°C under humid conditions. Embryos were staged *in ovo* according to Hamburger and Hamilton (Hamburger and Hamilton, 1951). Sexing was performed by PCR, as described previously (Clinton et al., 2001). ZW females were identified by the presence of a female-specific (W-linked) *XhoI* repeat sequence in addition to a *18S* ribosomal gene internal control. ZZ males showed the 18S band only (Clinton et al., 2001).

### Sex-reversal experiments

Sex reversal experiments were performed as described previously (Estermann et al., 2021a). Briefly E3.5 (HH stage 19) eggs were injected with 1.0 mg of Fadrozole (Novartis), PBS (Vehicle), 0.1mg of E2 in 10% ethanol in sesame oil solution or vehicle (control). Urogenital systems for WISH or left gonads for qRT-PCR were collected at E9.5 (HH35).

### Whole-mount *in situ* hybridization

Whole-mount *in situ* hybridization was performed as described previously (Estermann et al., 2020). Briefly urogenital systems were fixed overnight in 4% PFA in DEPC-PBS. After methanol dehydration and rehydration to PBTX (PBS+0.1% Triton X-1000), tissues were permeabilized in proteinase K 10 mg/ml for up to 2 hours. Tissues were briefly re-fixed and placed into pre-hybridization solution overnight at 65°C. *TOX3* (Estermann et al., 2020), *AMH* (Lambeth et al., 2015) and aromatase (Hirst et al., 2017) antisense probes were added to pre-hybridized tissues (∼7.5 µl/tube) and hybridization was carried out overnight at 65°C. Tissues were then subjected to stringency washes, blocked in TBTX/BSA/sheep serum and then treated overnight with anti-DIG-AP antibody (1:2000; Roche). After extensive washing in TBTX, tissues were exposed to BCIP/NBT color reaction at room temperature for up to 3 h. Color reaction was stopped at the same time for each gene by rinsing in NTMT buffer, TBTX, PBTX, PBS and imaging. To examine tissue sections, samples were overstained for 2 days, cryoprotected in PBS plus 30% sucrose, snap frozen in OCT and cryosectioned (10 µm).

### RNA extraction and qRT-PCR

Gonadal pairs were collected in 330 µl of Trizol reagent (ThermoFisher) and kept at −80°C until processing. After sexing, three same-sex gonadal pairs were pooled for each sample, homogenized and the RNA was extracted according to the manufacturer's instructions (Trizol, ThermoFisher). For chicken DF-1 cells, confluent cells on each well (24-well plate) were collected in 1 ml of Trizol reagent (ThermoFisher) and stored at −80°C until processing. Genomic DNA was removed using DNA-free DNA Removal Kit (Invitrogen) and 500 ng-1µg of RNA was converted into cDNA using Promega Reverse Transcription System (A3500). RT-qPCR was performed using QuantiNova SYBR Green PCR Kit. Primers used were: *TOX3* Fw, TCAGAGCTTGGATCTCCCCT; *TOX3* Rv, GGCGATACTGCGAAACTTGG; *SOX9* Fw, GTACCCGCATCTGCACAAC; *SOX9* Rv, TTCTCGCTCTCATTCAGCAG; *DMRT1* Fw, GGACTGCCAGTGCAAGAAGT; *DMRT1* Rv, GGTACAGGGTGGCTGATCC; *AMH* Fw, GAAGCATTTTGGGGACTGG; *AMH* Rv, GGGTGGTAGCAGAAGCTGAG; *β-actin* Fw, GCTACAGCTTCACCACCACA; *β-actin* Rv, TCTCCTGCTCGAAATCCAGT. Expression levels were quantified by the 2^−ΔΔCt^ method using β-actin as the housekeeping internal control gene. Data were analyzed using an unpaired *t*-test (two groups), multiple *t*-tests (one per embryonic stage/treatment) or one-way nonparametric ANOVA (if more than two groups were analyzed). Statistical significance was determined using the Holm-Sidak method for the multiple *t*-test or Tukey's test for ANOVA.

### Immunofluorescence

Embryonic urogenital systems were briefly fixed in 4% PFA/PBS, cryo-protected in 30% sucrose, blocked in OCT, snap frozen and 10 µm frozen sections were then cut. Immunofluorescence was carried out as described previously (Estermann et al., 2020). Briefly, sections were permeabilized in 1% Triton X-100 in PBS for 10 min at room temperature, blocked in 2% BSA/PBS for 1 h at room temperature followed by primary antibody incubation overnight at 4°C. The following primary antibodies were used: goat anti-GFP antibody (Rockland, 600-101-215, 1:500), mouse anti-pan cytokeratin (Novus Bio, NBP2-29429, 1:200), rabbit anti-DMRT1 (in-house antibody RRID AB_2665399; 1:2000), rabbit anti-SOX9 (Millipore antibody, AB5535, 1:4000), rabbit anti-AMH (Abexa, ABX132175, 1:1000), rabbit anti-aromatase (in-house antibody RRID AB_2734780; 1:5000), rabbit anti-CVH (in-house antibody; 1:500), rabbit anti-FOXL2 (in-house antibody; 1:2000), rabbit anti-p27 (Charles River Laboratories, 10100766, 1:1000) and mouse anti-TOX3 (Novus Bio, NBP2-45165, 1:100). Sections were then washed in PBS and incubated for 1 h at room temperature with Alexa Fluor 488 donkey anti-goat or rabbit (Life Technologies, A11055 and A21206, 1:1000) and Alexa Fluor 594 donkey anti-rabbit or mouse (Life Technologies, A21203 and A21207, 1:1000) diluted in 1% BSA/PBS. Sections were washed, counterstained with DAPI in PBS and mounted in Fluorsave (Millipore). Images were collected on a Zeiss Axiocam MRC5.

### Tissue section fluorescence *in situ* hybridization

*CYP17A1* fluorescence *in situ* hybridization in E9.5 gonadal paraffin wax-embedded sections was performed as described previously (Estermann et al., 2020). To colocalize the steroidogenic cell marker *CYP17A1* with AMH, aromatase or GFP, sections were subjected to antigen retrieval and then processed for immunofluorescence. We used goat anti-GFP (Rockland, 600-101-215, 1:500), rabbit anti-aromatase (in-house antibody RRID AB_2734780; 1:5000) or rabbit anti AMH (Abexa, ABX132175, 1:1000) primary antibodies with Alexa Fluor 488 donkey anti-rabbit or goat secondary antibody (Life Technologies, A11055 and A21206, 1:1000), followed by Sudan Black to quench cell autofluorescence. Sections were counterstained using DAPI in PBS and mounted in Fluorsave (Millipore).

### RCAS plasmid generation

*TOX3* and *SOX9* open reading frames were amplified from chicken gonadal cDNA and Gibson cloned into the *RCASBP(D)-GFP-T2A* and *RCASBP(B)* viral vectors, respectively. *RCASBP(D)-GFP-T2A-TOX3* and *RCASBP(B)-SOX9* plasmid sequences were confirmed by Sanger sequencing. Primers used can be found in Table S1.

Two different shRNAs (*sh370* and *sh685*) were designed against the *TOX3* ORF, ranked for effectiveness (Clarke et al., 2017) and cloned into the *RCASBP(A)-BFP* plasmid (Estermann et al., 2021a). Correct cloning and sequences were confirmed by Sanger sequencing. Primers used can be found in Table S1.

### DF-1 cell culture and transfection

DF-1 chicken fibroblastic cells were seeded onto 24-well plates and transfected with 1.5 µg of each construct according to the Lipofectamine 2000 protocol (Life Technologies). *RCASBP(A)-DMRT1* (Lambeth et al., 2014), *RCASBP(A)-SOX9* or *RCASBP(A)-GFP* plasmids were transfected in DF-1 cells in a 24-well plate. At 48 h post-transfection, cells were washed with 1×PBS, collected in 1 ml of TRIzol and stored at −80°C until processing. To validate the *TOX3*-overexpression plasmid, *RCASBP(D)-GFP-T2A-TOX3* or *RCASBP(A)-GFP* was transfected into DF-1 cells. At 48 h post-transfection cells were collected for RNA extraction or fixed for immunofluorescence against GFP and TOX3.

To test the shRNA, DF-1 cells plated in a 24 well plate were transfected with *RCASBP(A)-BFP-TOX3sh370*, *RCASBP(A)-BFP-TOX3sh685* or *RCASBP(A)-BFP-Firefly-Sh774* (non-silencing control) using Lipofectamine. After 48 h, cells were transfected with *RCASBP(D)-GFP-T2A-TOX3*. At 48 h post-transfection, cells were fixed briefly with 4% PFA in PBS and immunofluorescence against GFP was performed. To validate the ability of *TOX3 sh685* to knock down *TOX3* expression, T-25 flasks containing DF1 cells were transfected with *RCASBP(A)-BFP-TOX3sh685* or *RCASBP(A)-BFP-Firefly-Sh774* (non-silencing control). At 72 h post-transfection they were collected and plated in 24-well plates. After resting for 24 h, DF-1 cells were transfected with *RCASBP(D)-GFP-T2A-TOX3* overexpression plasmid. At 48 h post-transfection, cells were collected in 1 ml of Trizol reagent and processed for RNA extraction.

### Virus purification

RCAS virus propagation and purification was performed as reported previously (Smith et al., 2009b). Briefly, viral plasmids were introduced into small T-25 flasks containing DF-1 cells using Lipofectamine 2000 (Life Technologies). Cells were passaged into six T-175 flasks and grown until they were super-confluent. Media were replaced with 1% FCS DMEM and harvested over 3 consecutive days. Virus was concentrated by ultracentrifugation, resuspended in 600 µl and titered.

### *In vivo* overexpression and knock down experiments

For *DMRT1* knockdown experiments *RCASBP(B)-GFP-DMRT1shRNA343* or *RCASBP(B)-GFP-scrambled* (control) live virus was injected into 0-day chicken blastoderms, as previously described (Smith et al., 2009a). For *TOX3* knockdown experiments, *RCASBP(A)-BFP-TOX3sh685* or *RCASBP(A)-BFP-Firefly-Sh774* live virus was injected in 0-day chicken blastoderms. Embryos were collected at E9.5 in both cases.

*RCASBP(A)-DMRT1, RCASBP(A)-GFP or RCASBP(D)-GFP-T2A-TOX3* over-expression plasmids were electroporated *in ovo* into the left coelomic epithelium, as previously described (Lambeth et al., 2014). Embryos were collected at E7.5 for *DMRT1*-overexpression experiments or E9.5 for *TOX3*-overexpression experiments. The same method was used to introduce *RCASBP(A)-BFP-TOX3sh685* or *RCASBP(A)-BFP-Firefly-Sh774* knockdown constructs. Embryos were also collected at E9.5. TOX3 sh685 and control shRNA electroporation resulted in a survival rate of 25-30%. TOX3 overexpression and GFP control electroporation resulted in a survival rate of 25-30%.

## Supplementary Material

10.1242/develop.201466_sup1 Supplementary information
