# Supplementary material for: DMRT1-mediated regulation of TOX3 modulates expansion of the gonadal steroidogenic cell lineage in the chicken embryo
Source: Development. 2023 Mar 9;150(5):dev201466. doi: 10.1242/dev.201466 (PMC10108705; doi:10.1242/dev.201466)
Supplement: Supplementary file 1 [file develop-150-201466_review_history.pdf]

DMRT1 regulation of *TOX3* modulates expansion of the gonadal steroidogenic cell lineage in the chicken embryo

Martin Andres Estermann, Andrew Thomas Major and Craig A. Smith
DOI: 10.1242/dev.201466

Editor: Haruhiko Koseki

Review timeline

Original submission:	22 November 2022
Editorial decision:	28 December 2022
First revision received:	18 January 2023
Accepted:	25 January 2023

Original submission

First decision letter

MS ID#: DEVELOP/2022/201466

MS TITLE: DMRT1 regulation of TOX3 modulates expansion of the gonadal steroidogenic cell lineage

AUTHORS: Martin Andres Estermann, Andrew Thomas Major, and Craig A Smith

I have now received all the referees' reports on the above manuscript, and have reached a decision. The referees' comments are appended below, or you can access them online: please go to BenchPress and click on the 'Manuscripts with Decisions' queue in the Author Area.

As you will see, the referees express considerable interest in your work, but have some significant criticisms and recommend a substantial revision of your manuscript before we can consider publication. If you are able to revise the manuscript along the lines suggested, which may involve further experiments, I will be happy receive a revised version of the manuscript. Your revised paper will be re-reviewed by one or more of the original referees, and acceptance of your manuscript will depend on your addressing satisfactorily the reviewers' major concerns. Please also note that Development will normally permit only one round of major revision. If it would be helpful, you are welcome to contact us to discuss your revision in greater detail. Please send us a point-by-point response indicating your plans for addressing the referee's comments, and we will look over this and provide further guidance.

Please attend to all of the reviewers' comments and ensure that you clearly highlight all changes made in the revised manuscript. Please avoid using 'Tracked changes' in Word files as these are lost in PDF conversion. I should be grateful if you would also provide a point-by-point response detailing how you have dealt with the points raised by the reviewers in the 'Response to Reviewers' box. If you do not agree with any of their criticisms or suggestions please explain clearly why this is so.

Reviewer 1

Advance summary and potential significance to field

These researchers previously showed the surprising result that steroidogenic cells in the chicken gonad arise from differentiated supporting cells (Eastermann et al, Cell Reports 2020). In this manuscript, the team shows a credible mechanism to explain this phenomenon - induction of the

© 2023. Published by The Company of Biologists under the terms of the Creative Commons Attribution License (https://creativecommons.org/licenses/by/4.0/).

transcription factor TOX3 by the chicken male-determining factor DMRT1. The work is done mainly in the chicken embryonic testis, and demonstrates that TOX3 acts to suppress transition of embryonic Sertoli (supporting) cells to Leydig (steroidogenic) cells. TOX3 expression can be repressed by estrogens in vivo but its expression requires the dominant male DMRT1 factor. It is not clear whether TOX3 represses expansion of the steroidogenic lineage directly (via cell lineage allocation) or indirectly (by signaling from supporting to steoroidogenic cell populations). In my opinion this work makes a significant and novel contribution to our understanding of cell fate specification that will be of general interest to developmental biologists.

Comments for the author

Points to address:

The title should probably include the words 'in chicken'

Line 97 - from Figure 1E/F it appears that TOX3 is also highly expressed in the Leydig cell cluster? Do you need to comment on this and how does it fit with your IF data and hypothesis as to TOX3 function?

Line 107 - why do you think TOX3 only expressed in a subset of Sertoli cells (what is different about them?)

Line 116 - please define DF-1 cells (chicken embryonic fibroblast) - reader is not told this until line 259

Line 141 - when in vivo TOX3 knockdown is performed - SOX9 expression diminished, but this is not the case for DMRT1 or AMH. At the same time we see an increased number of CYP17A1+ Leydig cells, suggesting TOX3 is required to inhibit Leydig cell differentiation. Why do you think only SOX9 expression is diminished - why not other Sertoli cell markers DMRT1 and AMH?

Line 186 - It might be useful to defind 'intermediate cells' in the Introduction section, when you are explaining about the finding of steroidogenic cells being derived from differentiated supporting cells.

Line 194 - 'to evaluate the role of TOX3 in ovarian differentiation' seems a bit odd in terms of introduction to this section, because you have told us that TOX3 is not normally expressed in the developing chick ovary. So perhaps you should be 'evaluating the effect of ectopic expression of TOX3 in the developing ovary' (to determine if the repression of steroidogenic fate is a general phenomenon).

Line 204 - why do you think ectopic expresion of TOX3 in chicken fetal ovary leads to increased expression of AMH (but not female to male sex reversal, as would be indicated by SOX9 or DMRT1 increased expression)? This information about AMH upregulation is repeated at line 214 (although you are referring to a different figure).

Cortical (non-meduallary) is defined line 203, but this should be done earlier (line 198)

Throughout the manuscript, sometimes refer to CYP19A1 and sometimes aromatase (even in the same paragraph, eg from line 215 - 217)

I am curious as to why so few CYP17A1 positive cells are detected in E9.5 sections (Figure 2C)? Are there more if you look later?

The figures are not labelled (Fig1, Fig2 etc)

Figure 5 - panels not sufficiently labelled, sex not shown.

Figure 2 - the legend says IF is detecting P27 (infection marker) and TOX3, but the figure is labelled 'ShRNA' and 'TOX3'?? Why would P27 be an infection marker? I don't see any white arrows, just white arrow heads?

© 2023. Published by The Company of Biologists under the terms of the Creative Commons Attribution License (https://creativecommons.org/licenses/by/4.0/).

IMHO the conclusion section contains too much repetition of the results, though the potential relevance of these findings to the etiology PCOS is very interesting indeed.

Reviewer 2

Advance summary and potential significance to field

The somatic cell differentiation in early gonads, especially differentiation into supporting cells and steroidogenic cells, are key events for gonadal development. Authors have previously reported the important evidence that the coelomic epithelium gives rise to gonadal epithelium and interstitial cells and the supporting cells derive from pre-existing mesenchymal cell population in chicken embryo. These developmental origins are differed from mouse embryos, therefore to reveal the exact mechanism regulating of differentiation process is necessary for understanding Evo-devo of sex determination in birds. Authors previously identified the TOX3 expressed in embryonic Sertoli cells of the chicken testis. In this paper, TOX3 function is analyzed by taking multiple approaches, TOX3 knockdown, over-expression and mis-expression E2 exposure, aromatase inhibition, and DMRT1 over-expression and knockdown. The function of TOX3 proposed in this paper is supported by a huge set of data, with reasonable conclusions. however, there is little evidence for its regulation. I consider that if the suggested points are fully revised, this is suitable for publication in Development.

Comments for the author

- Relationship of DMRT1, SOX9 and AMH is confusing. In mammals, SOX9 directly regulates AMH, but in chicken, AMH regulation is independent of SOX9. To make the results easier to understand, how about mentioning the relationship and the timing of expression start of these genes including TOX3, in the part of "TOX3 expressed in a..." in Result section?

- What is the expression of TOX3 in tissues other than gonad? Could you show the results of WISH in whole embryo?

- Line 231-245. According to the theoretical order of conclusions, you should show the results of aromatase inhibition first and then the results of E2 exposed? Also, ABC and DEF in Fig6 are exchanged.

- Line 346-. Authors hypothesized the DMRT1 directly regulates TOX3 and SOX9 expression, however no direct evidence was given. Molecular biology experiments such as CHIP-seq or reporter gene assays should be performed to provide evidence. Alternatively, genome informatics tool should be used to demonstrate the presence of binding sites for DMRT1 in the regulatory regions of these genes.

- Line 368-373. The type of somatic cells that surround germ cells is a very important finding. JCM should be explained in more detail with reference.

Reviewer 3

Advance summary and potential significance to field

The manuscript: „ DMRT1 regulation of Tox3 modulates expansion of the gonadal steroidogenic cell lineage” provides new and valuable information on the transcriptional network of testis differentiation in chicken. Following previous investigations on DMRT1- dependent gonadal differentiation in chicken as a model of avian differentiation, the authors provide a next interesting piece of information on the regulatory network of especially the male steroidogenic cell lineage development.

Comments for the author

The manuscript: „ DMRT1 regulation of Tox3 modulates expansion of the gonadal steroidogenic cell lineage” provides valuable information on the transcriptional network of gonadal differentiation in

© 2023. Published by The Company of Biologists under the terms of the Creative Commons Attribution License (https://creativecommons.org/licenses/by/4.0/).

chicken. Following previous investigations on DMRT1- dependent gonadal differentiation in chicken as a model of avian differentiation, the authors provide a next interesting piece of information on the regulatory network of especially the steroidogenic cell lineage development. Based on the previous information for expression of Tox3 specifically in the developing gonads in chicken, the single-cell transcriptional sequencing was deepened, and Tox3 expression was shown to be restricted to the Sertoli cell lineage and Sertoli-Leydig cell cluster. The authors verified the initial single cell expression information with sex and development specific RT-PCR data. The localization patterns were moreover described on the transcriptional level using in-situ hybridization and at the protein level with immunohistochemical localization compared to Sertoli cell markers. Interestingly, DMRT1 is not expressed in the germ cell lineage at all.

The approach of local knock down of Tox3 applying two Tox3 shRNAs cloned into the RCASBP viral vector expressing BFP/GFP as reporter in cell culture to validate the expression of the constructs and thereafter in vivo provided specific and significant reduction of Tox3 RNA and protein for the Tox 3 sh685 construct in vitro and in-vivo. The same approach worked well for the overexpression experiment for Tox3 as well as for DMRT1.

The knockout and overexpression experiments could clearly demonstrate the influence of DMRT1 and Tox3 on the expression characteristics of the steroidogenic cells. However, in the methods section the number of embryos treated, and the survival rate to the different ages for analysis should be stated clearly for each experiment. For demonstration of exclusive integration of constructs electroporated specifically into the left body side, the overview in Fig.2 panel A would be more informative showing the gonads of both body sides In the overview.

In Fig.2A the label of the third panel ShRNA is questioned. In the Figure legend the interferon marker P27 is named.

It is not clear, why panels B and C were not arranged as panel A starting with the overlay of the lower magnification and adding the overview of the higher magnification followed by the single channel images at high magnification. Moreover, the in-situ hybridization data in Fig. 2C need single channel data with higher magnification to be unequivocally visible. The same holds true for Fig.3. The single channel images would provide better information form magnified frames. Panels B and C would be better adjusted to the same magnification as in panel A.

It is not conclusive why Fig. 4 includes Two magnification levels for Tox3 labeling, but none for GFP alone.

In Fig. 5 the panels should be labeled and the single channel images might be better exchanged by the higher magnification. The white arrows mentioned n the Figure legends to indicate steroidogenic cells are missing.

Interesting are the experiments with E2 and fadrozole treatments of the embryos and the induced reaction of Tox3 and steroidogenic markers. The downregulation of Tox3 after E2 induction was clearly demonstrated in males with corresponding repression of the Sertoli cell markers and compared to the missing upregulation of Tox3 in females after fadrozole treatment although aromatase, the female specific steroidogenic marker was downregulated. This effect undermines the importance of DMRT1 as testis specifying master gene. The Tox3 downregulation followed by Sox9 and AMH reduction and aromatase and Foxl2 upregulation in DMRT1-blocked gonads after DMRT1 shRNA transfection further undermined this initiation function. In Fig. 8 the panel should be labeled. The frames for indicating the transfected cell region do not really ease the Figure and might be omitted.

Altogether, these experiments provide a very interesting information on the regulatory network of gonadal differentiation in chicken.

After editing the minor issues mentioned, the manuscript could be recommended for publication.

© 2023. Published by The Company of Biologists under the terms of the Creative Commons Attribution License (https://creativecommons.org/licenses/by/4.0/).

First revision

Author response to reviewers' comments

Reviewer 1 Advance Summary and Potential Significance to Field...

These researchers previously showed the surprising result that steroidogenic cells in the chicken gonad arise from differentiated supporting cells (Eastermann et al, Cell Reports 2020). In this manuscript, the team shows a credible mechanism to explain this phenomenon- induction of the transcription factor TOX3 by the chicken male-determining factor DMRT1.
….In my opinion this work makes a significant and novel contribution to our understanding of cell fate specification that will be of general interest to developmental biologists.

Points to address:

The title should probably include the words 'in chicken'

RESPONSE: Corrected. See new title.

Line 97 - from Figure 1E/F it appears that TOX3 is also highly expressed in the Leydig cell cluster? Do you need to comment on this and how does it fit with your IF data and hypothesis as to TOX3 function?

RESPONSE: Leydig cells were actually a small TOX3 negative group of cells clustering closely with a subset of Sertoli cells, not initially clear on our figure 1. Fig 1E and F have been revised to make this clearer. In our initial single-cell RNA-seq, containing both male and female datasets of we were not able to identify a distinct Leydig cluster per se. Instead, we were able to identify male and female steroidogenic cells clustering together in what we defined as the steroidogenic cell cluster. Fig 1E and Fig 1F show this for male cells only, on t-SNE plots. The Leydig cells are shown in burgundy color on a revised Fig 1E. Interestingly, these cells clustered among as a subset Sertoli cells, lime green on revised fig 1E. As can be seen in figure 1F, *TOX3* is not expressed in the Leydig cells (burgundy), but in the Sertoli cells in the small sub-cluster (lime green). Additionally, in Supplementary figure 2C and D, TOX3 is not expressed in the cells expressing the highest levels of CYP17A1 (Leydig cells). This could suggest that TOX3 might be expressed in those early intermediate Sertoli cells that are potentially transitioning towards steroidogenic cells (double positive cells), but it is turned off once the cells commit into the Leydig cell fate. We corrected the text accordingly. See lines 101-102

Line 107 - why do you think TOX3 only expressed in a subset of Sertoli cells (what is different about them?)

RESPONSE: This is an interesting question. In chicken, the Sertoli cell population may not be homogenous. Two clear examples are the presence of SOX9+ AMH negative cells in the testis, or the expression of the testis factor HEMGN in some but not all Sertoli cells (Lambeth 2014). The protein expression of TOX3 also agrees with this idea of a more complex Sertoli cell population. With the recent findings regarding Sertoli and Leydig cell differentiation, our hypothesis is that the cells that express TOX3 are the cells that will maintain their Sertoli cell identity, whereas the TOX3 negative are the ones that will transition into Leydig cells. This hypothesis is supported by the overexpression and knock down experiments. We do not know the exact mechanism that generates this diversity of Sertoli cell fates.

Line 116 - please define DF-1 cells (chicken embryonic fibroblast) - reader is not told this until line 259

RESPONSE: Corrected and defined. Line 120

Line 141 - when in vivo TOX3 knockdown is performed - SOX9 expression diminished, but this is not the case for DMRT1 or AMH. At the same time we see an increased number of CYP17A1+ Leydig cells, suggesting TOX3 is required to inhibit Leydig cell differentiation. Why do you think only SOX9 expression is diminished - why not other Sertoli cell markers DMRT1 and AMH?

© 2023. Published by The Company of Biologists under the terms of the Creative Commons Attribution License (https://creativecommons.org/licenses/by/4.0/).

RESPONSE: DMRT1 and AMH gene expression begin before that of SOX9 and TOX3 in embryonic chicken testis. Simplistically, the temporal sequence is DMRT1 – AMH – SOX9/TOX3. Hence, TOX3 knockdown might be expected to affect SOX9 but not upstream regulators such as DMRT1. DMRT1 was shown by our laboratory to be necessary and sufficient to induce SOX9 expression. In this manuscript we demonstrate that TOX3 is a downstream factor of DMRT1. This suggests that both TOX3 and SOX9 are under the regulation of DMRT1. In addition, TOX3 and SOX9 have a similar expression timeframe. mRNA expression of both genes can be detected at E6.5, but the protein expression is detected later in development. Our results show that when TOX3 is knocked down, SOX9 is also downregulated, suggesting that TOX3 might participate in regulating SOX9 expression. But TOX3 is not the only factor regulating SOX9, as it fails to induce SOX9 expression in the overexpression experiments. This could be explained by the lack of DMRT1 expression, suggesting that TOX3 and DMRT1 (and may be other factors) are required to induce SOX9 expression. We now included a summary figure (Figure 9) where we show the role of TOX3 in the context of gonadal sex differentiation.

Line 186 - It might be useful to defind 'intermediate cells' in the Introduction section, when you are explaining about the finding of steroidogenic cells being derived from differentiated supporting cells.

RESPONSE: We know defined the intermediate cells in the introduction as suggested. See lines 50-51.

Line 194 - 'to evaluate the role of TOX3 in ovarian differentiation' seems a bit odd in terms of introduction to this section, because you have told us that TOX3 is not normally expressed in the developing chick ovary. So perhaps you should be 'evaluating the effect of ectopic expression of TOX3 in the developing ovary' (to determine if the repression of steroidogenic fate is a general phenomenon).

RESPONSE: Yes, text corrected as suggested. Lines 196-198

Line 204 - why do you think ectopic expresion of TOX3 in chicken fetal ovary leads to increased expression of AMH (but not female to male sex reversal, as would be indicated by SOX9 or DMRT1 increased expression)? This information about AMH upregulation is repeated at line 214 (although you are referring to a different figure).

RESPONSE: As stated in the discussion, estrogen and AMH are mutually antagonistic in the avian gonad. Suppression of aromatase, and hence estrogen synthesis, allows up-regulation of AMH expression in the female gonad. Conversely, exogenous estrogen (E2, 17-β-estradiol) causes AMH mRNA down-regulation in male gonads. Mis-expression of TOX3 in female gonads had an effect upon the endocrine development of the gonad, by locally activating AMH and suppressing aromatase expression. As TOX3 knockdown in males did not affect AMH expression, it is likely that ectopic TOX3 in the female gonad affects AMH expression indirectly by suppressing aromatase expression. In the male gonad, one of the roles of TOX3 in the supporting cell lineage (Sertoli cells) may be to ensure that the aromatase gene is inactive, hence preventing feminization. It would be of interest to scrutinize the CYP19A1 (Aromatase) gene for TOX3 binding sites.
Regarding the repetition, figures 4 and 5 are both study the role of TOX3 overexpression in ovaries. Figure 4 uses immunofluorescence to determine the expression of different gonadal markers, whereas figure 5 uses fluorescent in situ hybridization to determine steroidogenic cell differentiation. In both figures, aromatase and AMH are evaluated. In figure 4 to evaluate the colocalization of the overexpressing construct and AMH and Aromatase and in figure 5 to evaluate the co-expression of AMH or Aromatase with CYP17A1 (double positive/intermediate population). Because these are two different techniques and derive from different electroporated gonads, we were stating that the phenotype was consistent among samples and techniques. We now included some extra description regarding figure 5 in the text (Lines 218-220).

Cortical (non-meduallary) is defined line 203, but this should be done earlier (line 198)

RESOPONSE: Manuscript corrected as suggested (Line 202)

© 2023. Published by The Company of Biologists under the terms of the Creative Commons Attribution License (https://creativecommons.org/licenses/by/4.0/).

Throughout the manuscript, sometimes refer to CYP19A1 and sometimes aromatase (even in the same paragraph, eg from line 215 - 217)

RESPONSE: *CYP19A1* is the gene, Aromatase is the gene product. For consistency, we have generally used “Aromatase”. (line 221).

I am curious as to why so few CYP17A1 positive cells are detected in E9.5 sections (Figure 2C)? Are there more if you look later?

RESPONSE: This may be a timing issue, and yes, there may be more CYP14A1 cells if we were to examine later stages. Our previous studies have shown that differentiation of fetal Leydig cells is a relatively late phenomenon compared to that in mouse. Unfortunately, we do not typically evaluate later timepoints due to animal ethics constraints. (Animal ethics approval would be required in our institute for such chicken experiments beyond mid- gestation). It would be interesting to evaluate how plastic is the steroidogenic cell line when significantly reduced, especially if they proliferate more after or if they produce more androgens to compensate the low number of steroidogenic cells.

The figures are not labelled (Fig1, Fig2 etc)

RESPONSE: Now the figures are labelled.

Figure 5 - panels not sufficiently labelled, sex not shown.

RESPONSE: Figure 5 now corrected.

Figure 2 - the legend says IF is detecting P27 (infection marker) and TOX3, but the figure is labelled 'ShRNA' and 'TOX3'?? Why would P27 be an infection marker? I don't see any white arrows, just white arrow heads?

RESPONSE: shRNA should be named “P27” on the figure. This is now corrected. P27 shows robust viral delivery. RCAS virus P27 is a virus protein, and we use immunofluorescence to detect it and hence report the successful spread of the viral vector after electroporation. Note, this is not the p27 that is associated with cell cycle. This is now clearly stated on Fig 2 legend.

IMHO the conclusion section contains too much repetition of the results, though the potential relevance of these findings to the etiology PCOS is very interesting indeed.

RESPONSE: Conclusion section has been reduced.

Reviewer 2 Advance Summary and Potential Significance to Field...

… In this paper, TOX3 function is analyzed by taking multiple approaches, TOX3 knockdown, over-expression and mis-expression, E2 exposure, aromatase inhibition, and DMRT1 over- expression and knockdown. The function of TOX3 proposed in this paper is supported by a huge set of data, with reasonable conclusions. however, there is little evidence for its regulation. I consider that if the suggested points are fully revised, this is suitable for publication in Development.

Reviewer 2 Comments for the Author...

- Relationship of DMRT1, SOX9 and AMH is confusing. In mammals, SOX9 directly regulates AMH, but in chicken, AMH regulation is independent of SOX9. To make the results easier to understand, how about mentioning the relationship and the timing of expression start of these genes including TOX3, in the part of "TOX3 expressed in a..." in Result section?

RESOPONSE: Yes, the onset of AMH expression precedes that of SOX9 in birds. This is now noted in Results section (lines 80-85 of revised text) and as part of a new summary figure (Figure 9).

© 2023. Published by The Company of Biologists under the terms of the Creative Commons Attribution License (https://creativecommons.org/licenses/by/4.0/).

- What is the expression of TOX3 in tissues other than gonad? Could you show the results of WISH in whole embryo?

RESPONSE: TOX3 is tightly developmentally regulated, being expressed in specific tissues during chicken embryogenesis. We performed whole mount in situ hybridization in E4.5 chicken embryos and found similar expression patterns to what was reported in E14.5 mouse embryo (Sahu SK et al. doi: 10.1016/j.bbagrm.2016.04.005) Particularly we found *TOX3* mRNA expression in the forebrain, the neural tube, the otic vesicle, as well as fainter in both the forelimb and hindlimb (Left). Shown below. In the middle and right panels, we show a section of TOX3 WISH on a E4.5 chicken embryo, showing positive expression in the dorsal region of the neural tube, (but no expression yet in the gonads).

E4.5 TOX3 WISH

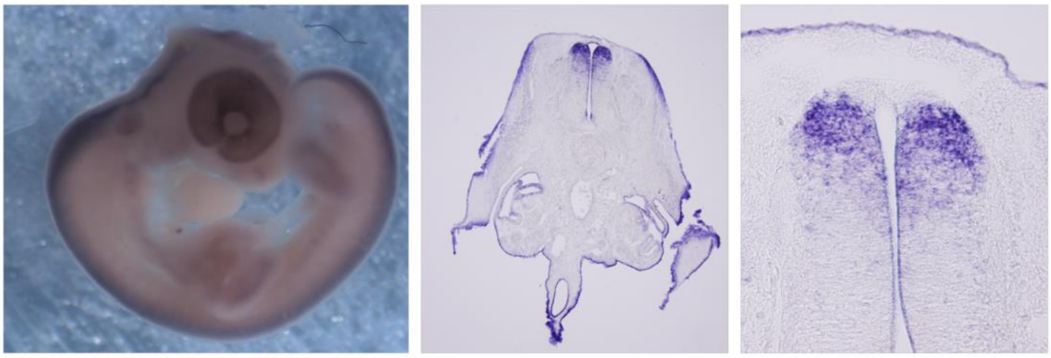

- Line 231-245. According to the theoretical order of conclusions, you should show the results of aromatase inhibition first and then the results of E2 exposed? Also, ABC and DEF in Fig6 are exchanged.

RESPONSE: We prefer to keep this order. We start with the positive data, where exposure to E2 retards TOX3 expression male gonads. Then estrogen inhibition results (Fadrozle treatment) show that the absence of estrogens is not sufficient to induce *TOX3* expression, suggesting the involvement of other masculinizing factors. This factor was explored in figures 7 and 8, where we identified DMRT1 as the necessary and sufficient factor to induce TOX3 expression. This best fits of the flow, where failure of estrogen inhibition to have an effect on TOX3 leads to the conclusion that other factors are needed to trigger TOX3, and this then leads into the regulation of TOX3 by DMRT1 in the next section.

- Line 346-. Authors hypothesized the DMRT1 directly regulates TOX3 and SOX9 expression, however no direct evidence was given. Molecular biology experiments such as CHIP-seq or reporter gene assays should be performed to provide evidence. Alternatively, genome informatics tool should be used to demonstrate the presence of binding sites for DMRT1 in the regulatory regions of these genes.

RESPONSE: We used the word hypothesis instead of claiming to be a direct role because we do not have the sufficient evidence to test that hypothesis. We agree with the reviewer that DMRT1 ChIP-seq would be crucial to test if DMRT1 directly or indirectly regulates *TOX3* expression. We decided to remove that phrase from the discussion to avoid confusion.

- Line 368-373. The type of somatic cells that surround germ cells is a very important finding. JCM should be explained in more detail with reference.

RESPONSE: We now include a definition and explanation for the JCM, (the juxtacortical medulla) as suggested. (Lines 350-354)

Reviewer 3 Advance Summary and Potential Significance to Field...

The manuscript: „ DMRT1 regulation of Tox3 modulates expansion of the gonadal steroidogenic cell lineage" provides new and valuable information on the transcriptional network of testis

© 2023. Published by The Company of Biologists under the terms of the Creative Commons Attribution License (https://creativecommons.org/licenses/by/4.0/).

differentiation in chicken. Following previous investigations on DMRT1- dependent gonadal differentiation in chicken as a model of avian differentiation, the authors provide a next interesting piece of information on the regulatory network of especially the male steroidogenic cell lineage development.
Altogether, these experiments provide a very interesting information on the regulatory network of gonadal differentiation in chicken. After editing the minor issues mentioned, the manuscript could be recommended for publication.

Reviewer 3 Comments for the Author...

However, in the methods section the number of embryos treated, and the survival rate to the different ages for analysis should be stated clearly for each experiment.

RESPONSE: In TOX3 knock down experiment by electroporation, the survival rates in both the control and the knock down were around 25-30%. TOX3 overexpression experiments resulted 20-25% survival rate. We included that data in the methodology. See lines 560-562

For demonstration of exclusive integration of constructs electroporated specifically into the left body side

RESPONSE: The left lateral electroporation always results in left side only electroporation. An example for the shRNA electroporation is now showing in the Supp figure 1C.

the overview in Fig.2 panel A would be more informative showing the gonads of both body sides In the overview

RESPONSE: The right side from the same embryo is not electroporated and we do not show these in our manuscript. Only the left sides are typically kept for immuno- analysis, hence we do not harvest the right-side tissue. We need to convincingly show high power views of the left electroporated gonads. To show both left and right would mean lower resolution and loss of the information we wish to convey. More importantly, the best control is not a right gonad but the left gonad of other embryos that have been electroporated but with viral plasmids expressing GFP or a non-silencing shRNA.

In Fig.2A the label of the third panel ShRNA is questioned. In the Figure legend the interferon marker P27 is named.

RESPONSE: The same question was asked by reviewer 1 and is answered above. The third panel should read “P27” not shRNA, as it shows staining for P27. We now correctly label this. Note that, here, P27 marks a virus epitope (not interferon, nor a cell cycle marker which have same acronyms). RCAS virus P27 is a virus protein, and we use immunofluorescence to detect it and hence report the successful spread of the viral vector after electroporation.

It is not clear, why panels B and C were not arranged as panel A starting with the overlay of the lower magnification and adding the overview of the higher magnification followed by the single channel images at high magnification.

RESPONSE: This figure is now corrected to show the magnification overlay as well as the individual channels.

Moreover, the in-situ hybridization data in Fig. 2C need single channel data with higher magnification to be unequivocally visible.

RESPONSE: Similarly, this figure is now corrected to show the magnification overlay as well as the individual channels.

The same holds true for Fig.3. The single channel images would provide better information form magnified frames. Panels B and C would be better adjusted to the same magnification as in panel A.

© 2023. Published by The Company of Biologists under the terms of the Creative Commons Attribution License (https://creativecommons.org/licenses/by/4.0/).

RESPONSE: As above, Figure 3 is now corrected to show the magnification overlay as well as the individual channels.

It is not conclusive why Fig. 4 includes Two magnification levels for Tox3 labeling, but none for GFP alone.

RESPONSE: This Figure is now corrected to show the magnification overlay as well as the individual channels to keep it consistent with the previous images.

In Fig. 5 the panels should be labeled and the single channel images might be better exchanged by the higher magnification. The white arrows mentioned n the Figure legends to indicate steroidogenic cells are missing.

RESPONSE: Figure is corrected to show the magnification overlay as well as the individual channels. We now included the sex and white and blue arrows for steroidogenic and transitioning cells.

Interesting are the experiments with E2 and fadrozole treatments of the embryos and the induced reaction of Tox3 and steroidogenic markers. The downregulation of Tox3 after E2 induction was clearly demonstrated in males with corresponding repression of the Sertoli cell markers and compared to the missing upregulation of Tox3 in females after fadrozole treatment although aromatase, the female specific steroidogenic marker was downregulated. This effect undermines the importance of DMRT1 as testis specifying master gene. The Tox3 down-regulation followed by Sox9 and AMH reduction and aromatase and Foxl2 upregulation in DMRT1-blocked gonads after DMRT1 shRNA transfection further undermined this initiation function.

RESPONSE: This series of experiments examined the response of TOX3 to estrogen manipulation, and the main conclusion was that estrogen can act to keep TOX3 expression repressed (when it is added to male, TOX3 goes down). The estrogen data do not say anything directly about DMRT1 and the data does not undermine DMRT1 as the master testis gene. However, the effects of exogenous estrogen most likely reflect its antagonism against DMRT1, as we showed previously, Smith et al. 2003. Biology of Reproduction, 68; 560-570 (and more recently by others: doi.org/10.1101/2022.02.22.481469. We know that DMRT1 definitely is the master testis gene, as recently shown by DMRT1 CRISPR/Cas9 DMRT1 monoallelic knockout chickens, which form ovaries, not testes (Ionnidas et al. PNAS, 2021).

In Fig. 8 the panel should be labeled. The frames for indicating the transfected cell region do not really ease the Figure and might be omitted.

RESPONSE: When infecting gonads with viral vector that deliver shRNAs, delivery variable within and between gonads. It is therefore important to highlight those regions of the gonads that have robust delivery. This is what the frames (dotted lines) depict. This is important, as it focus the attention of the viewer to the region where shRNA was successfully delivered in the supporting cells. In this case, localized DMRT1 knockdown, which matched activation of female markers (Aromatase and FOXL2) and loss of male markers (DMRT1 itself, TOX3, SOX9 and AMH).

Second decision letter

MS ID#: DEVELOP/2022/201466

MS TITLE: DMRT1 regulation of TOX3 modulates expansion of the gonadal steroidogenic cell lineage in the chicken embryo

AUTHORS: Martin Andres Estermann, Andrew Thomas Major, and Craig A Smith
ARTICLE TYPE: Research Article

I am happy to tell you that your manuscript has been accepted for publication in Development, pending our standard ethics checks.

© 2023. Published by The Company of Biologists under the terms of the Creative Commons Attribution License (https://creativecommons.org/licenses/by/4.0/).

Reviewer 2

Advance summary and potential significance to field

The somatic cell differentiation into supporting cells and steroidogenic cells in early embryo are key events for gonadal development in vertebrate. Authors have previously reported the important evidence that the coelomic epithelium gives rise to gonadal epithelium and interstitial cells and the supporting cells derive from pre-existing mesenchymal cell population in chicken embryo. These developmental origins are differed from mammal (mouse) embryos, therefore to reveal the exact mechanism regulating of differentiation process is necessary for understanding of gonadal development in birds. This paper can provide a model in which TOX3 modulates the steroidogenic cell population differentiation, and its dysregulation may underlie increased steroidogenic capacity, leading to understanding of a human disease, PCOS.

Comments for the author

Thank you for responding to my comments. The authors have responded satisfactorily to requests from reviewers and the manuscript is well revised. I consider this paper to be of sufficient quality for publication. I look forward to further research progress in TOX3 function.

Reviewer 3

Advance summary and potential significance to field

As already stated for the original manuscript: „ DMRT1 regulation of Tox3 modulates expansion of the gonadal steroidogenic cell lineage” provides novel and valuable information on the transcriptional network of gonadal differentiation in chicken. Following previous investigations on DMRT1- dependent gonadal differentiation in chicken as a model of avian differentiation, the authors provide a next interesting piece of information on the regulatory network of especially the steroidogenic cell lineage development. Based on the previous information for expression of Tox3 specifically in the developing gonads in chicken, the single-cell transcriptional sequencing was deepened, and Tox3 expression was shown to be restricted to the Sertoli cell lineage and Sertoli-Leydig cell cluster. The authors verified the initial single cell expression information with sex and development specific RT-PCR data. The localization patterns were moreover described on the transcriptional level using in-situ hybridization and at the protein level with immunohistochemical localization compared to Sertoli cell markers. Interestingly, DMRT1 is not expressed in the germ cell lineage at all and exclusively activated for somatic gonadal differentiation.

Comments for the author

The revised version of the manuscript fulfills the required minor adaptations.

Thus, the report: „ DMRT1 regulation of Tox3 modulates expansion of the gonadal steroidogenic cell lineage” is recommended for publication in the current state.

© 2023. Published by The Company of Biologists under the terms of the Creative Commons Attribution License (https://creativecommons.org/licenses/by/4.0/).
